# Moisture-Curable Poly(hydroxyurethanes) Composites Reinforced with Silica Nanofillers

**DOI:** 10.3390/polym18172061

**Published:** 2026-08-25

**Authors:** Theo Bride, Eline Laurent, Jad Raad, Milan Marić

**Affiliations:** Department of Chemical Engineering, McGill University, Montreal, QC H3A 0C5, Canada; theo.bride@mail.mcgill.ca (T.B.); or eline.laurent@cttei.com (E.L.); or jadraad0@gmail.com (J.R.)

**Keywords:** Non-Isocyanate Polyurethanes, Silica, Hybrid Polyhydroxyurethanes, silica-based hybrids, moisture-curing

## Abstract

Despite safety advantages over conventional polyurethanes, one of the biggest drawbacks of polyhydroxyurethanes (PHUs) are their comparatively weak mechanical properties derived from their limited molecular weights via poly(addition). Improvement of mechanical properties of a neat, moisture-curable PHU, synthesized from typical diamine and dicarbonate precursors, was attained by blending both fumed silica and amino-functionalized silica, independently. Varying filler loadings, and both amine-terminated and cyclic carbonate-terminated PHU prepolymers (synthesized in bulk using telechelic amino-terminated poly(propylene glycol), number average molecular weight M_n_~2000 g mol^−1^) and diglycerol dicarbonate, resulted in a much-improved formulation in terms of hardness, modulus, and tensile properties. The amine-terminated prepolymer cross-linked with 3-glycidyloxypropyltrimethoxysilane (GLYMO) and 3.5 wt% of fumed silica had the highest hardness, 2.01 MPa, and the highest modulus, 5.07 MPa, according to nanoindentation tests, and we obtained E of 0.0280 MPa, σ_u_ of 0.118 MPa, and ε_u_ of 13.2%, all of which were unobtainable for the benchmark PHU as it was mechanically too weak to perform the tests. All samples were successfully cross-linked, and we found that those reinforced with amino-functionalized silica were weaker than their fumed silica counterparts due to the formation of aggregates, which were confirmed using SEM and TEM imaging.

## 1. Introduction

Over the past two decades, increasing attention has focused on developing alternatives to conventional polyurethanes (PUs), resulting in the emergence of nonisocyanate polyurethanes (NIPUs). PUs, the sixth most produced polymer globally [1], are highly versatile and used in coatings, adhesives, elastomers, sealants, thermoplastics, and foams [2]. Despite their low cost and effectiveness, concerns about PU’s impact on human health and the environmental have emerged. The diisocyanates commonly used in PU synthesis, such as methylene diisocyanate (MDI) and toluene diisocyanate (TDI), are classified as carcinogenic, mutagenic, and reprotoxic (CMR) under European REACH and the Globally Harmonized System [3]. Moreover, the production of isocyanates requires specialized handling of highly toxic phosgene gas [4].

A common route to NIPUs is the polyaddition of a five-membered cyclic carbonate with a diamine, yielding polyhydroxyurethanes (PHUs) [2]. This process eliminates the use of phosgene and diisocyanates while still forming urethane linkages containing additional hydroxyl groups. These hydroxyl groups introduce more extensive hydrogen bonding, which both enhance adhesion properties but also impede reaction kinetics and thus limit the synthesis of high-molecular-weight, linear PHUs [5].

One strategy to overcome these limitations is to cross-link, chain-extend, or branch PHUs, thereby increasing their overall molecular weight and improving mechanical strength, often by using hybridization methods. Hybrid PHUs can be synthesized by incorporating organic or inorganic species in the cross-linking of PHU, for example, [6,7,8,9]. A common approach is to employ the sol–gel process [9,10], in which PHUs are end-capped with silane to produce moisture-curable materials. In this process, ambient moisture first hydrolyzes the silane end groups to silanols, which subsequently condense to form a siloxane network, yielding a cross-linked hybrid material.

Previous work in our group has demonstrated the efficacy of this method in synthesizing hybrid PHUs derived from sugar-based cyclic carbonates [8,11,12,13,14]. Besides employing cross-linking, the addition of fillers offers a route to improve mechanical properties. The empirical framework below is derived and expands on the baseline model developed by Bride (2025) in the thesis entitled “Incorporation of Inorganic and Organic Nanoparticles Within Moisture Curable Polyhydroxyurethane Matrices” [15]. In this study, we report the synthesis of hybrid PHUs using the sol–gel process with the addition of nanofillers. Although there have been multiple studies investigating amine or cyclic carbonate-functionalized silica nanofillers in PHUs [7,9], and many studies investigating moisture curing silica agents in PHUs [9], to the best of our knowledge, there have not been any studies investigating the reinforcement of PHUs through both silica-based moisture curing agents and amine-functionalized silica nanofillers simultaneously.

Several silica-based fillers have been applied to enhance PUs. Fumed silica (FS), a widely used relatively inexpensive inorganic filler, has been extensively investigated in conventional polyurethanes. Zhou et al. demonstrated that both hydrophilic and hydrophobic FS increased surface roughness, micro-indentation hardness, and elastic modulus, as well as the force required to fracture an acrylic-based PU [16]. Akram et al. showed that incorporating 2 wt% FS into linseed oil-based PUs optimized scratch hardness, corrosion resistance, and thermal stability [17,18].

In contrast, amino-functionalized silica (AS), although less common industrially, can establish covalent bonds with the polymer. Wu et al. reported that 3 wt% AS in PUs tripled tensile strength and increased thermal degradation temperature relative to neat PU [6]. Chen et al. also investigated hydroxyl-functionalized, superhydrophobic, and amino-functionalized silicas in PHUs. All fillers enhanced rubbery plateau moduli (10–29%), tensile strength (7–38%), and strain at break (up to 18%) [7]. However, samples with reactive AS exhibited loss in the rubbery plateau storage modulus (E′) values after reprocessing. This is attributed to imperfect network formation and disruption of the stoichiometric amine balance, as well as a decrease in cross-link density during remolding.

Polyhedral oligomeric silsequioxanes (POSS) can act as both filler and cross-linker. MacInnis et al. capped cyclic carbonate-terminated PHUs with aminopropyl-POSS and 3-isocyanatopropyltrimethoxysilane, observing up to a 100% increase in modulus, a 77% increase in tensile strength, and 27% increase in elongation at break [8]. Similarly, Blattmann and Mülhaupt carbonated epoxy-POSS and cured it with hexamethylenediamine to produce PHUs with 20–45× higher modulus and up to 2.7× greater tensile strength than non-POSS PHUs [19]. Furthermore, silanes such as [3-(2,3-epoxypropoxy)-propyl]-trimethoxysilane (GLYMO) and N-(2-aminoethyl)-3-aminopropyltrimethoxysilane (DAMO) have been employed as moisture-curable cross-linkers in sol–gel processes [8,11,12,13]. End-capping PHUs with these silanes yields siloxane networks after condensation. In some applications, however, POSS’s costs may be prohibitive.

**Figure 1:**
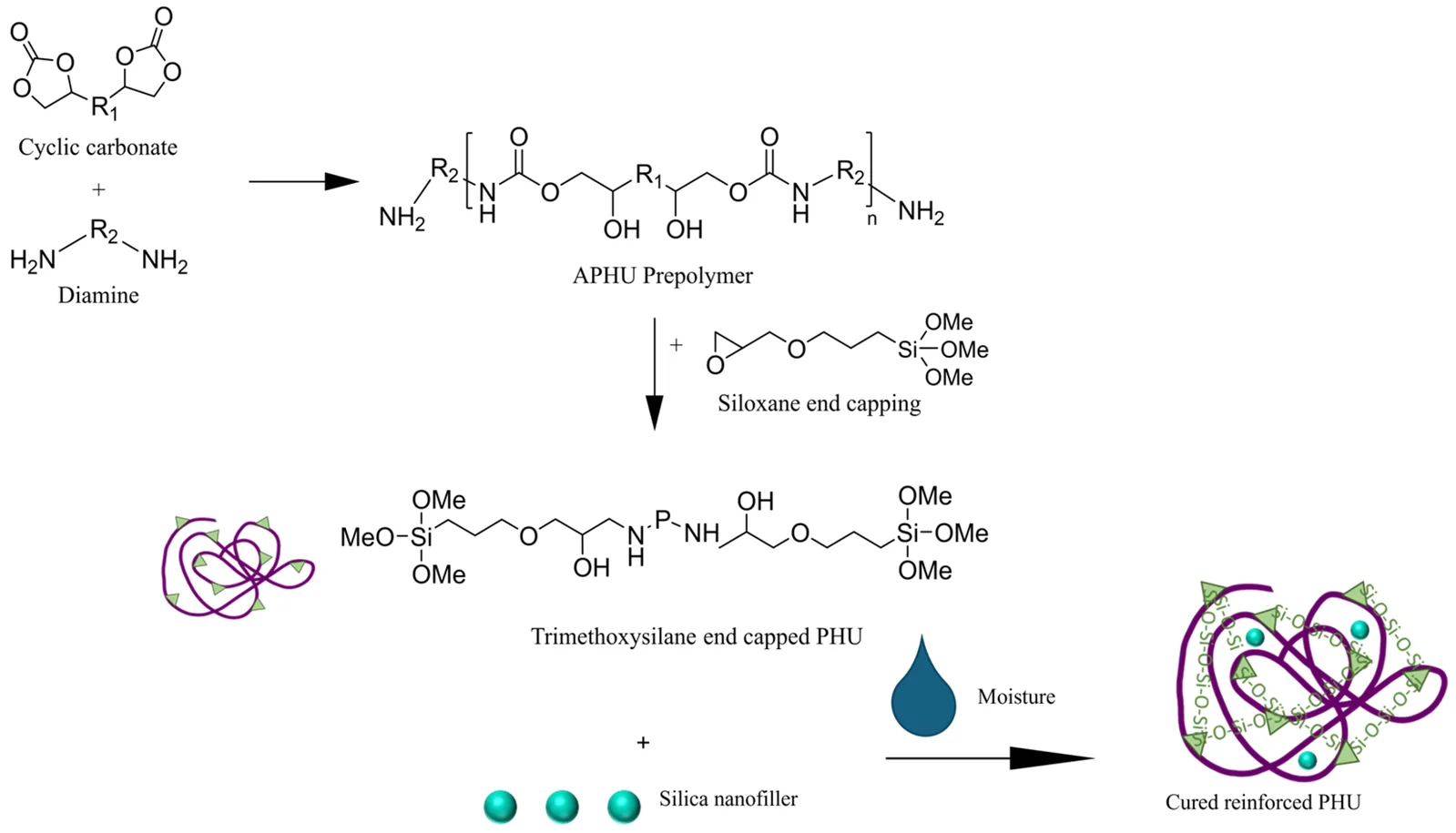
Overview of all synthesis steps. Reproduced with permission from Theo Bride, Incorporation of Inorganic and Organic Nanoparticles Within Moisture Curable Polyhydroxyurethane Matrices; published by McGill Libraries, 2025 [15].

Thus, we compare the effect of silica-based fillers like FS and AS on PHU mechanical properties while using GLYMO and DAMO to facilitate cross-linking of the reinforced PHU. Specifically, we prepared hybrid PHUs via the polycondensation of diglycerol carbonate (DGC), a bio-derived cyclic carbonate, and Jeffamine D-2000, a commercially available telechelic amine-terminated poly(propylene glycol). GLYMO and DAMO, commonly employed as moisture-curable cross-linkers in sol–gel processes [8,11,12,13], were chosen to limit the use of catalysts and high temperatures [11]. The resulting prepolymer was reinforced with silica nanofillers and cured under ambient conditions with a silane curing agent. A schematic of the proposed synthetic route is shown in Figure 1.

## 2. Materials and Methods

### 2.1. Materials

Diglycerol (DIG, ≥80% α,α, impurities consist of mono-, α,β-di, β,β-di, and triglycerol) was obtained from TCI. Dimethyl carbonate (DMC, ≥99%, anhydrous), Jeffamine D-2000 (M_n_ = 2000 g/mol), deuterared chloroform (CDCl_3_, ≥99.8%), phenyl isocyanate (≥98%), benzophenone (≥99%), fumed silica (powder, 0.2–0.3 microns), 3-Glycidyloxypropyltrimethoxysilane (GLYMO), N-2-Aminoethyl-3-aminopropyltrimethoxysilane (DAMO), and triethylamine (≥99.5%) were purchased from Sigma-Aldrich. Ethyl acetate (EtOAc, ≥99.5%), toluene (≥99.5%), and tetrahydrofuran (THF, ≥99.9%) were purchased from Thermo Fisher Scientific. Potassium carbonate (used in the synthesis of DGC, ≥99%) was purchased from EMD Millipore. Reverse osmosis (RO) purified water was provided by McGill’s Chemical Engineering Department.

### 2.2. Characterization Methods

#### 2.2.1. Differential Scanning Calorimetry (DSC)

DSC tests were performed on a TA Instruments Discovery 2500 DSC (TA Instruments, New Castle, DE, USA). All samples were heated from 25 °C to 120 °C at a rate of 10 °C/min to erase the thermal history of the sample, then cooled to −90 °C at a rate of 10 °C/min, and finally heated again until 120 °C at a rate of 10 °C/min. The glass transition temperature (*T*_*g*_) of the samples was evaluated on the second heating ramp using the glass transition analysis tool present in the TRIOS software (version 5.1.4672). The TA Instruments TRIOS software was used to extrapolate the glass temperature during the second heating ramp.

#### 2.2.2. Electron Microscopy

Before performing any tests, the samples had to be frozen due to their low T_g_ and were microtomed for TEM analysis. The samples were sectioned with a Leica Microsystems EM UC7/FC7 cryo-ultramicrotome (Leica Microsystems, Wetzlar, Germany) with a Diatome diamond knife (Diatome, Nidau, Switerzerland) at −120 °C.

##### Scattering Electron Microscopy (SEM)

The flat surfaces of the sample blocks remaining after cryo-sectioning were coated with a 4 nm thick layer of platinum to enhance electrical conductivity and then imaged by the FEI Quanta 450 FE-SEM microscope (FEI, Hillsboro, OR, USA) operating at an accelerating voltage of 2 kV, spot size of 2.5, and working distance of 4 mm in secondary electron (SE) mode with an Everhart–Thornley SE Detector (ETD).

##### Transmission Electron Microscopy (TEM)

For TEM, the ultrathin sections of ~50 to 70 nm thickness of the samples were transferred onto 200-mesh Cu TEM grids with Formvar support film with 2.3 M sucrose in 0.1 M phosphate buffer. The grids containing the sections were washed carefully with distilled water to remove sugar solution and then dried. The grids were then imaged by a Thermo Scientific Talos F200X G2 S/TEM (Thermo Fisher Scientific, Waltham, MA, USA) equipped with a X-FEG High Brightness Schottky Field Emission Source and a Ceta 16 M 4 k × 4 k CMOS Camera at an accelerating voltage of 200 keV.

#### 2.2.3. Fourier Transfer Infrared Spectroscopy (FTIR)

FTIR measurements were performed using a Thermo Scientific Nicolet iS50 FTIR Spectrometer (Thermo Fisher Scientific, Waltham, MA, USA) equipped with a single-bounce diamond attenuated transmission reflectance and the OMNIC software (version 9.6.238). After obtaining a baseline reading by scanning the clean diamond, each sample was scanned 32 times over the range of 400–4000 cm^−1^ with a standard resolution of 4 cm^−1^.

#### 2.2.4. ^1^H Nuclear Magnetic Resonance Spectroscopy (NMR)

NMR spectra were obtained using a Bruker 500 MHz NMR spectrometer (Bruker Corporation, Billerica, MA, USA) at a temperature of 25 °C with 32 scans for the nuclei. For other variables such as relaxing time, the default values were used and are noted in Table S2. The PHU samples were dissolved in CDCl_3_ and analyzed using the MestReNova software (version 14.2.3-29241).

#### 2.2.5. Tensile Testing

For tensile testing, 3 samples were prepared in silicon molds shaped like tensile bars similar to those used in ASTM D638. The width, length, and thickness of the middle section of the bar were measured and inputted in the WinAGS Lite software (version 1.05). Once clamped to a Shimadzu EZTest Tensile Testing machine, the sample was pulled apart at a rate of 10 mm/min.

#### 2.2.6. Gel Content and Swelling Tests

Gel content tests and swelling tests were performed using THF, toluene, and RO-purified water. For each composite and each solvent, three samples of 30–50 mg (initial weight) were submerged in a glass vial with 5 mL of the corresponding solvent for 24 h. Each sample’s weight (swelled weight) was then measured before being put in a vacuum oven at 40 °C overnight (~18 h). After the sample was fully dry, its weight was measured again (dry weight). There was no difference between the samples submerged for 24 h and 48 h. Furthermore, there was no difference between the samples under vacuum for 18 h and 5 days.

The gel content (GC) was calculated as follows:

(1) $$GC\;(\%)=\frac{dried\;weight}{inital\;weight}\;\times \;100\%$$

The swelling index (SI) was calculated as follows:

(2) $$SI\;\left(\%\right)=\frac{swelled\;weight-initial\;weight}{initial\;weight}\;\times \;100\%$$

#### 2.2.7. Nanoindentation

Nanoindentation tests were performed using a Nanovea M1 Hardness Tester instrument (Nanovea, Inc., Irvine, CA, USA) equipped with a Berkovich tip. Three different locations, separated from each other by at least 1 mm, were tested for each sample. The approach speed was 1 μm/min with a contact load of 0.3 mN. Once that load was reached, a loading rate of 40 mN/min was applied, followed by a pause of 30 s to measure the creep, and then an unloading rate of 20 mN/min. The hardness and elastic modulus were calculated using the NMHTS Nanovea software (version 1.7.0).

#### 2.2.8. Thermogravimetric Analysis (TGA)

TGA tests were performed using a TA Instrument Discovery 5500 machine (TA Instruments, New Castle, DE, USA). All samples were heated from 25 °C to 600 °C at a heating rate of 10 °C/min on a platinum pan. The TA Instruments TRIOS software was used to extrapolate the onset degradation temperature (T_d5%_).

#### 2.2.9. Gel Permeation Chromatography (GPC)

The weight average molecular weight (*M*_w_), the number average molecular weight (*M*_*n*_), and the dispersity (Đ = *M*_w_/*M*_*n*_) of the prepolymer PHUs were measured using an Agilent 1260 Infinity II SEC (Agilent Technologies, Santa Clara, CA, USA). The samples were prepared with a concentration of 2–4 mg/mL. The columns were heated to 40 °C and HPLC-grade THF was used as an eluent at a flow rate of 0.3 mL/min. The injection volume was 10 μL and the elution time was 35 min for each sample. The GPC system was equipped with a PolyPore guard column (50 × 4.6 mm), an Agilent 1260 Infinity II (G7800A) Refractive Index (RI) detector (Agilent Technologies, Santa Clara, CA, USA), and two PolyPore columns (250 × 4.6 mm). M_n_ values were determined by calibration relative to linear narrow molecular weight distribution PMMA standards (Agilent Technologies, molecular weights ranging from 573 to 1,636,320 g/mol) and using the Agilent GPC software (version 2.2). Amine-containing species were quenched with phenyl isocyanate to prevent the polymers from absorbing onto the column packing.

#### 2.2.10. Rheological Measurements

The steady shear viscosity tests were performed using an Anton Paar Instruments MCR 302 rheometer (Anton Paar, Graz, Austria) using the RheoCompass software (version 1.23.403-Release). All tests were performed using the 25 mm parallel plate geometry. The viscosity versus time test was done under a shear rate ranging from 0.1 s^−1^ to 10 s^−1^ at ambient temperature.

### 2.3. Experimental Methods

#### 2.3.1. DGC and PHU Prepolymer Synthesis

**Figure 2:**
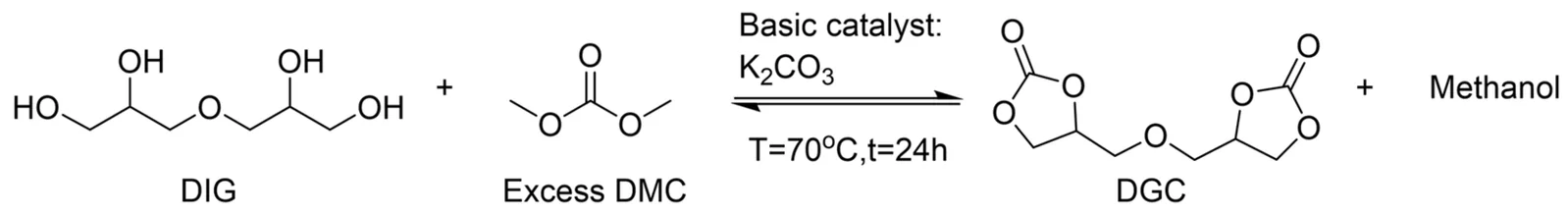
Reaction synthesis for DGC. Reproduced with permission from Theo Bride, Incorporation of Inorganic and Organic Nanoparticles Within Moisture Curable Polyhydroxyurethane Matrices; published by McGill Libraries, 2025 [15].

Before synthesizing the amine- or cyclic carbonate-terminated PHUs, diglycerol dicarbonate (DGC) was prepared by reacting diglycerol with dimethyl carbonate [15]. The cyclic carbonate product was then purified and characterized from an improved methodology described by Younes et al. [12]. The synthesis route is illustrated in Figure 2.

**Figure 3:**
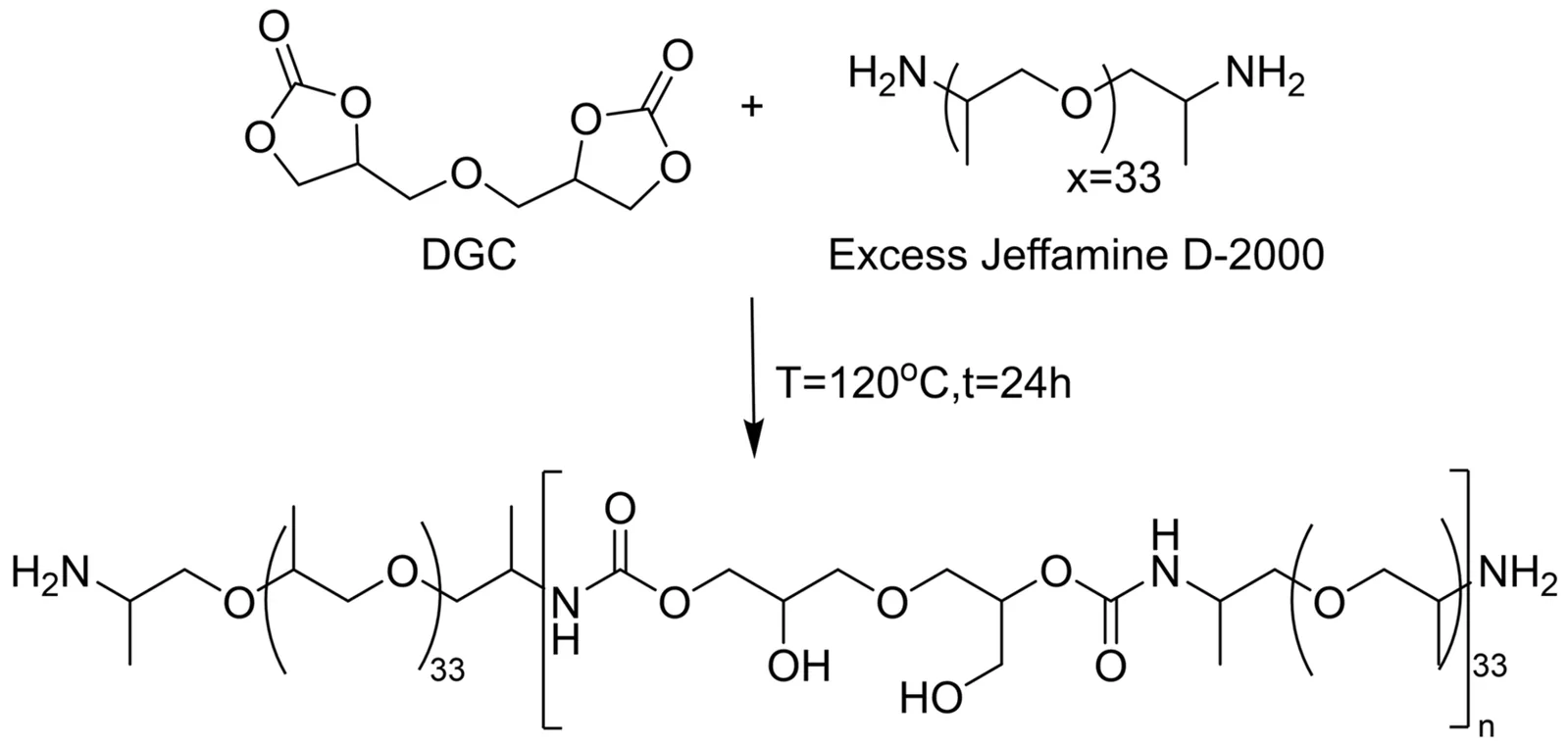
Reaction synthesis of amine-terminated PHU. Reproduced with permission from Theo Bride, Incorporation of Inorganic and Organic Nanoparticles Within Moisture Curable Polyhydroxyurethane Matrices; published by McGill Libraries, 2025 [15].

Subsequently, PHU prepolymers were synthesized in bulk via the polyaddition of DGC with Jeffamine D-2000. To obtain amine-terminated PHUs (APHU), Jeffamine D-2000 was used in excess at a 1:0.8 molar ratio (Jeffamine:DGC), whereas cyclic carbonate-terminated PHUs (CPHU) were produced by employing excess DGC (0.8:1 molar ratio) [15]. The reaction was carried out in a three-neck round-bottom flask equipped with a magnetic stirrer. The system was purged with nitrogen gas for 15 min at 3.5 kPa and then heated to 120 °C for 24 h in an oil bath, which was placed on a stir plate [15]. The reaction scheme for amine-terminated PHUs is shown in Figure 3; a similar procedure was used for the cyclic carbonate-terminated PHUs, except that the stoichiometry was altered to give cyclic carbonate end groups.

While the synthesis of amine-terminated PHUs proceeded readily, preparation of cyclic carbonate-terminated PHUs proved more challenging. Jeffamine’s reduced reactivity could be attributed to the steric hindrance exerted by the neighboring ether groups, which obstructs the amine group’s accessibility and reactivity [20,21]. Initial attempts under identical conditions (24 h at 120 °C) yielded only 50% conversion. As such, the temperature was increased to 140 °C, and after 25.5 h, the conversion still only reached 70%, despite using a 1:0.8 molar excess of DGC:Jeffamine. Since NMR analysis indicated residual amine end groups in the prepolymer, an additional 25 wt% of DGC was introduced to fully cap these species. This adjustment increased the overall conversion to 85%, producing CPHU with approximately 90% cyclic carbonate termination. Excess DGC was subsequently removed according to the purification procedure described by Farkhondehnia et al. [22].

#### 2.3.2. GLYMO PHU Composite

Prior to synthesizing the composites, amino-functionalized silica nanoparticles were prepared according to the method described by Ruiz-Cañas et al. [23]. In light of the considerably larger size of AS (160 nm) in comparison to FS (8–30 nm), we decided to incorporate both fillers as weight percent instead of mole percent to maintain a consistent volumetric fraction of filler relative to the polymer network. To produce PHU composites reinforced with either fumed or amino-functionalized silica, 1, 3.5, or 6 wt% filler was first dispersed in 10 mL ethyl acetate using an ultrasonic bath [15]. Dispersion required ~15 min for fumed silica but up to 90 min for amino-functionalized silica, depending on the filler loading. Once dispersed, 2.0 g of amine-terminated PHU was added and sonicated for a further 15 min. Next, 4 mol% triethylamine (2.1 µL) was introduced, and nitrogen was bubbled through the mixture for 30 min at 3.5 kPa to minimize moisture ingress prior to adding the curing agent. The nitrogen was then continuously bubbled throughout the reaction. GLYMO was added at 2 mol per mol of amine (0.35 g per mol), and the mixture was heated at 60 °C for 2 h in an oil bath. The mixture was subsequently poured into a silicone mold to allow for ethyl acetate evaporation and ambient moisture curing. After one week, the samples were heated at 80 °C for 4 h to complete curing, according to a previous study on a similar system [11].

**Figure 4:**
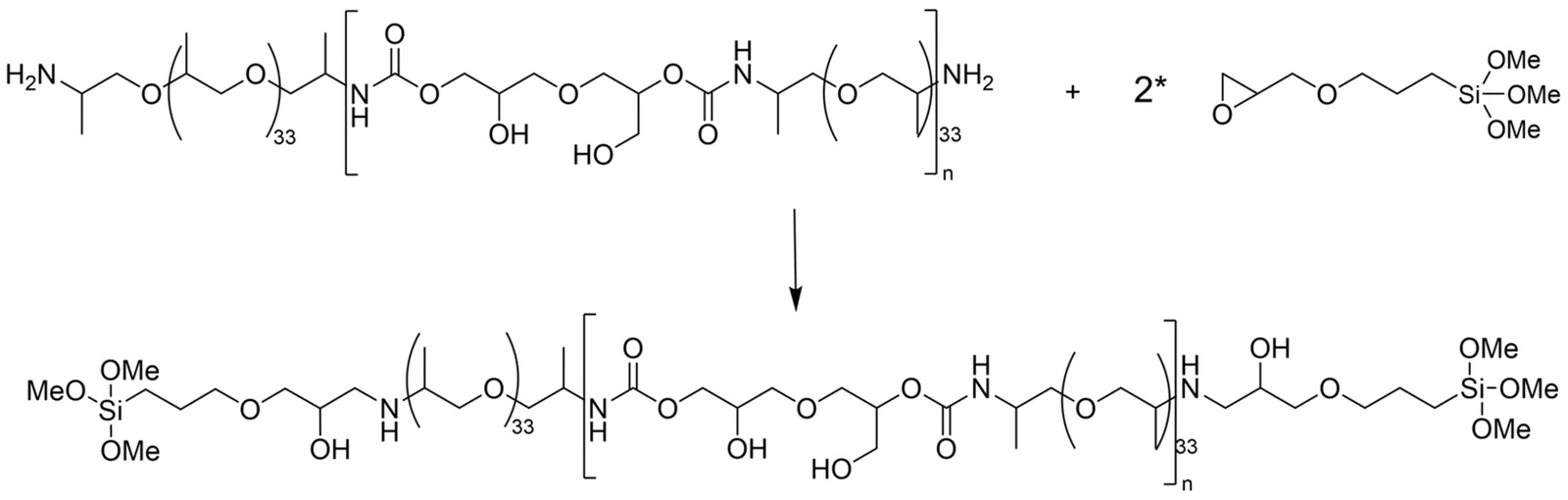
Capping reaction of APHU with GLYMO ratio of 2.0. The * symbol is a multiplication sign.

**Figure 5:**
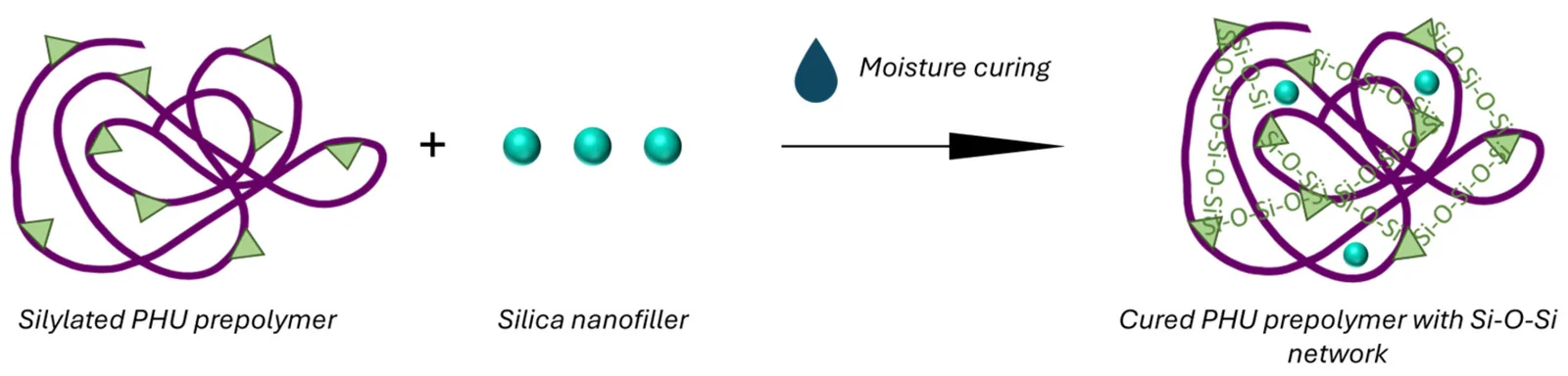
Moisture curing of GLYMO-capped PHU prepolymer with silica nanoparticles. Note that the fumed silica nanofillers may possess a native layer of OH groups due to oxidation.

Only a 2:1 GLYMO:amine molar ratio was tested, as per Younes et al. [11]. Figure 4 shows the capping of amine-terminated PHU with GLYMO, and the scheme for PHU composites containing silica nanoparticles is presented in Figure 5.

#### 2.3.3. DAMO PHU Composite

The DAMO PHU composites were synthesized by the same procedure as the GLYMO PHU composites, except that a cyclic carbonate-terminated PHU prepolymer was used.

## 3. Results and Discussion

### 3.1. Synthesis of PHU Prepolymer

Two PHU prepolymers were synthesized: one amine-terminated (APHU) and one cyclic carbonate-terminated (CPHU). Both were characterized by NMR, GPC, and FTIR, prior to the filler addition and curing reactions. The viscosity of the APHU can be found in Figure S15.

#### 3.1.1. NMR Characterization

**Figure 6:**
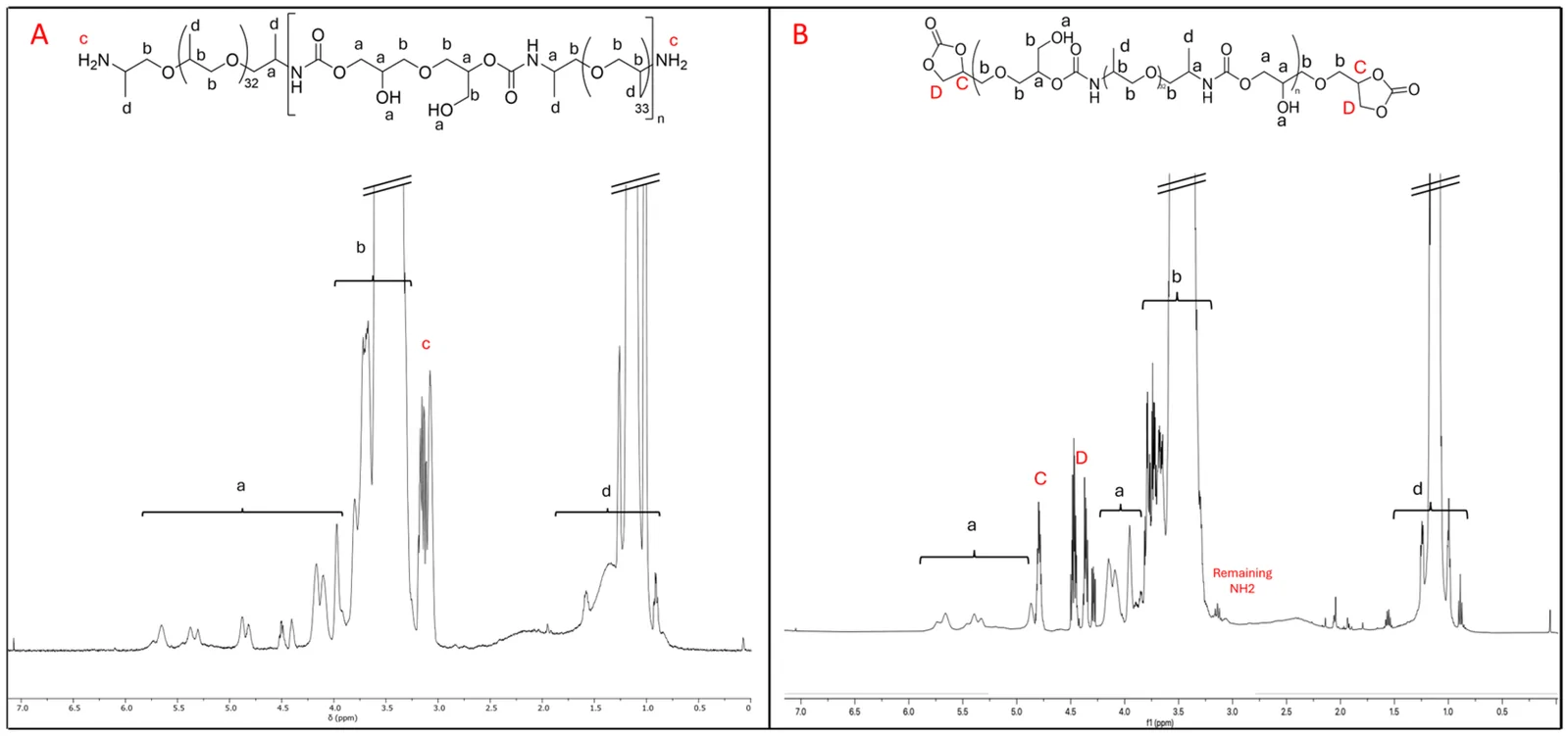
^1^H NMR of APHU (**A**) and CPHU (**B**) prepolymers dissolved in CDCl_3_.

NMR was employed to determine the number average absolute molecular weight of the PHU prepolymer, a key parameter for calculating the molar concentration of amine or cyclic functional groups and thus the required stoichiometry for the moisture curing agent for proper cross-linking. The NMR parameters such as relaxation time are tabulated in Table S2. The NMR spectra obtained at the end of the polyaddition reaction are shown in Figure 6.

Figure 6A shows no cyclic carbonates in the APHU prepolymer in the 4.0–6.0 ppm range, confirming that only amine groups remain at the polymer chain ends. In contrast, the CPHU retains some amine groups; analysis of three samples revealed an average of 10% amine functionality, with the remaining 90% being cyclic carbonates. The absolute molecular weights of the PHUs were determined using benzophenone as a marker, following the NMR Molecular Weight Calculation Procedure outlined in the supporting information. The APHU had an average molecular weight of 5400 g/mol, while the CPHU measured 3200 g/mol. These average molecular weights suggest that the APHU and CPHU are composed of trimers (Jeffamine-DGC-Jeffamine and DGC-Jeffamine-DGC) and pentamers (Jeffamine-DGC-Jeffamine-DGC-Jeffamine and DGC-Jeffamine-DGC-Jeffamine-DGC).

#### 3.1.2. GPC Characterization

**Figure 7:**
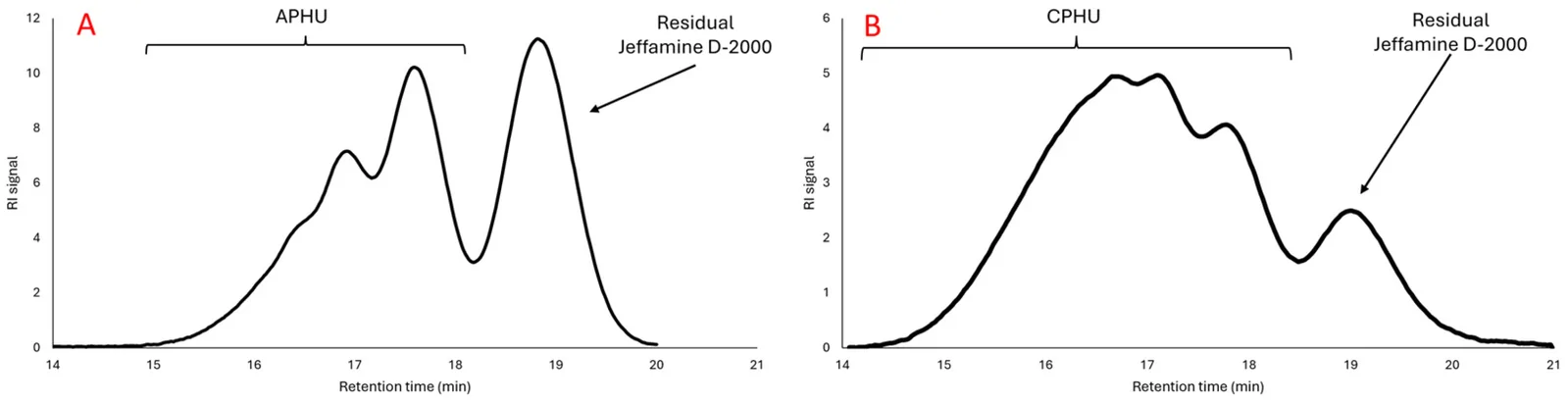
GPC trace of APHU (**A**) and CPHU (**B**).

GPC was used to obtain the M_n_ and M_w_ of the PHU prepolymer and to analyze the oligomer distribution. Data for APHU and CPHU are presented in Figure 7. The PHU prepolymers are multi-modal with several peaks due to the low degree of polymerization, which results in several distinct oligomers.

For the APHU (Figure 7A), polymers eluting between 15 and 18 min exhibited M_n_ = 8900 g/mol, M_w_ = 10,800 g/mol, and Đ = 1.21. Compounds eluting after 18 min showed M_n_ = 3100 g/mol, M_w_ = 3300 g/mol, and Đ = 1.045, corresponding to unreacted Jeffamine D-2000 (Figure S16). Note that the Đ of these individual peaks are slightly higher as the tails of each species overlap. Combined, these segments yield M_n_ = 5100 g/mol, M_w_ = 7800 g/mol, and Đ = 1.52. Note that the APHU sample was capped with phenyl isocyanate; thus, values may differ slightly for non-capped APHU prepolymer. For the CPHU (Figure 7B), polymers eluting between 14 and 21 min exhibited a M_n_ of 8700 g/mol, a M_w_ of 15,600 g/mol, and a Đ of 1.79. These values are relative to PMMA standards. The residual Jeffamine in the APHU originates from the excess amine relative to cyclic carbonate once the reaction was complete, while the residual Jeffamine in the CPHU comes from the incomplete reaction between the Jeffamine and DGC, despite the functional group imbalance.

#### 3.1.3. FTIR Characterization

**Figure 8:**
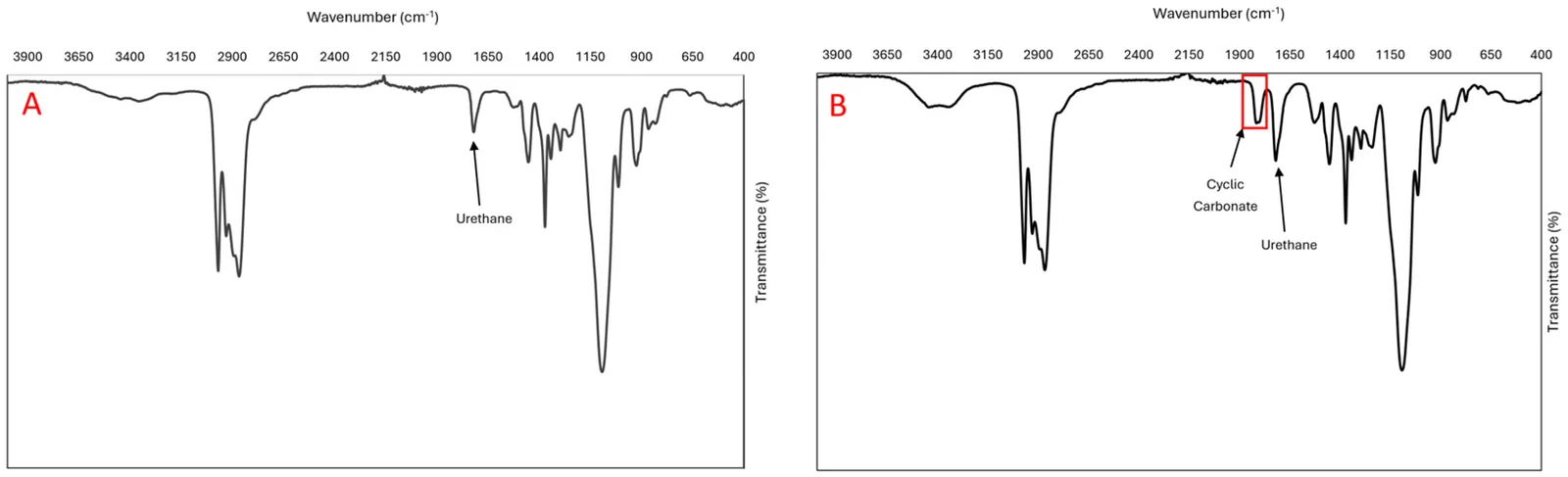
FTIR graph of APHU prepolymer (**A**) and CPHU prepolymer (**B**).

FTIR was employed to confirm the presence or absence of cyclic carbonate groups at the prepolymer chain ends [15]. However, it could not reliably detect the absence of amine groups due to overlapping NH (from the urethane) and NH_2_ signals, which remain equally concentrated throughout the polyaddition reaction. In Figure 8, the peak at 1790 cm^−1^ corresponds to the cyclic carbonate C=O bond; thus, this peak should vanish in amine-terminated prepolymers but persist in cyclic carbonate-terminated ones. Peak intensity correlates with the presence of cyclic carbonates.

Figure 8A shows the absence of the cyclic carbonate peak at 1790 cm^−1^, confirming that the prepolymer is amine-terminated. In contrast, the presence of this peak in Figure 8B indicates that the prepolymer is cyclic carbonate-terminated.

### 3.2. Synthesis of PHU Composites

#### GLYMO/DAMO PHU Composites

The hybrid PHUs were synthesized by following the steps outlined in the Experimental Methods Section. The samples are listed in Table 1.

### 3.3. Characterization of PHU Composites

The nomenclature for the GLYMO and DAMO composites is listed below in Table 1.

#### 3.3.1. FTIR

**Figure 9:**
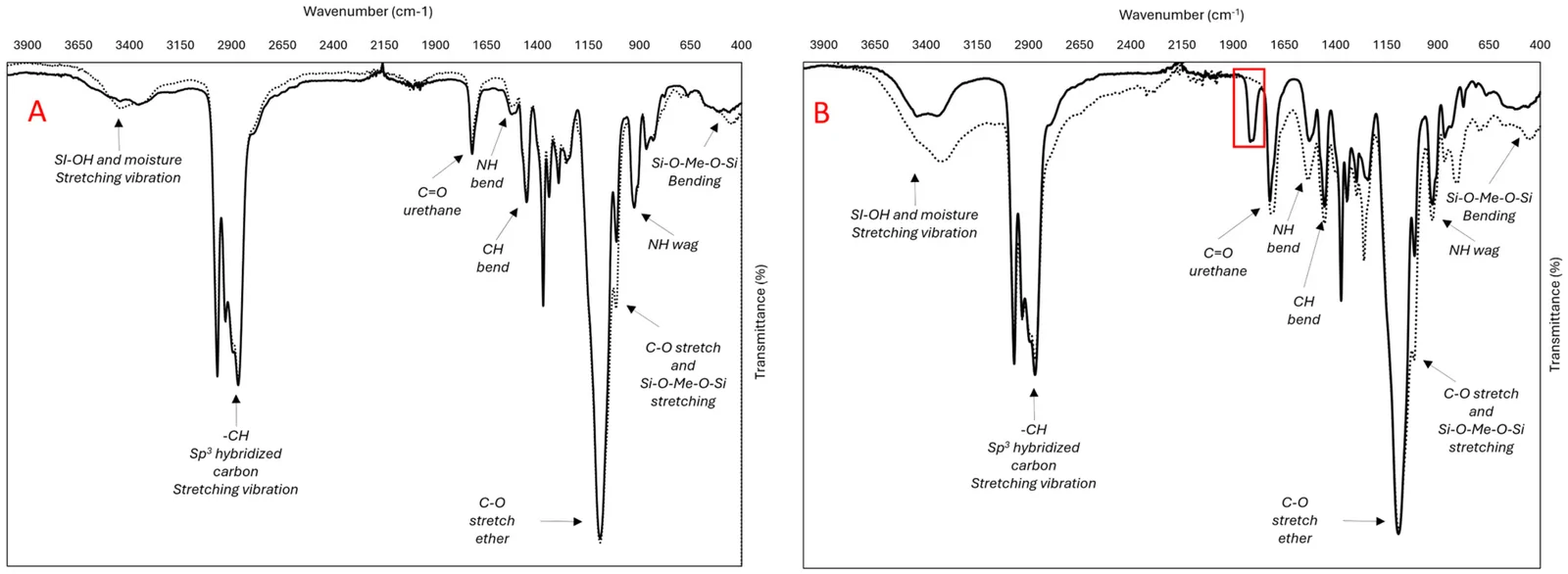
Stacked FTIR spectrum of APHU prepolymer (full) and 0wt% cured sample (dashed) (**A**), and stacked FTIR spectrum of CPHU prepolymer (full) and 0wt% cured sample (dashed) (**B**). The NH bend comes from the amine reacting with cyclic carbonates or epoxies. The Si-O-Me-O-Si come from the GLYMO and DAMO agents.

For the GLYMO composites, FTIR tracked the disappearance of the NH_2_ peak alongside the emergence of the Si–O–Me–O–Si bond [11]. The mechanism of the bond is shown in Figure S17. Figure 9 overlays the FTIR spectra of the amine-terminated prepolymer and the GLYMO composite without filler.

The increased peak absorption at 1000 cm^−1^ and the band at 480 cm^−1^, corresponding to Si-O-Me-O-Si bonds, confirm the formation of the inorganic network. Additionally, capping of CPHU with DAMO is evidenced by the complete disappearance of the cyclic carbonate peak at 1790 cm^−1^ (Figure 9B).

#### 3.3.2. Cross-Linking Characterization

After confirming the successful cross-linking, gel content tests were conducted to evaluate the extent of cross-linking. Swelling tests were then conducted to evaluate the cross-linking density and interaction of each sample with different solvents (RO water and toluene).

##### Gel Content

The gel content data collected is shown in Table 2.

All gel contents exceeded 60% across solvents, confirming successful cross-linking in all samples, consistent with previous PHU studies [11,24,25]. Gel content was consistently higher in water (87–98%) than in toluene (64–86%), likely due to the hydrophobic Jeffamine D-2000 in the PHU backbone, which would restrict water penetration and dissolution of unreacted compounds; this relative hydrophobicity also influences swelling behavior [26,27]. GLYMO-capped samples exhibited higher gel content than DAMO counterparts regardless of solvent (5–10% higher in water and 10–20% higher in toluene), indicating less effective cross-linking in CPHU samples. This is consistent with NMR data which showed incomplete cyclic termination and more unreacted chain ends. For gel content, no clear trends were observed regarding filler type or concentration.

##### Swelling Index

The swelling index data collected is shown in Table 3.

Generally, higher swelling indices indicate lower cross-linking densities and/or greater solvent affinity. Consistent with gel content results, samples submerged in water exhibited significantly lower swelling (5–75%) than those in toluene (90–230%), attributable to the hydrophobic nature of Jeffamine D-2000 present in the PHU backbone, which limits water uptake. Notably, GLYMO composites showed remarkably lower swelling in water (5–11%) compared to DAMO composites (46–75%). This discrepancy may be attributable to the reduced Jeffamine loading present within the DAMO composites, which was synthesized using the cyclic carbonate-terminated prepolymers. This difference in swelling within water can also stem from residual amine groups present within the CPHU not reacting with DAMO, which decrease the cross-linking density, and thus permit greater swelling despite lower solvent affinity. The reduced reactivity of cyclic carbonates with amines at room temperature could also have been preventing the CPHU from reacting with the DAMO [28]. Similarly to the gel content data, filler type and loading showed no clear effect on swelling behavior.

#### 3.3.3. DSC and TGA

**Figure 10:**
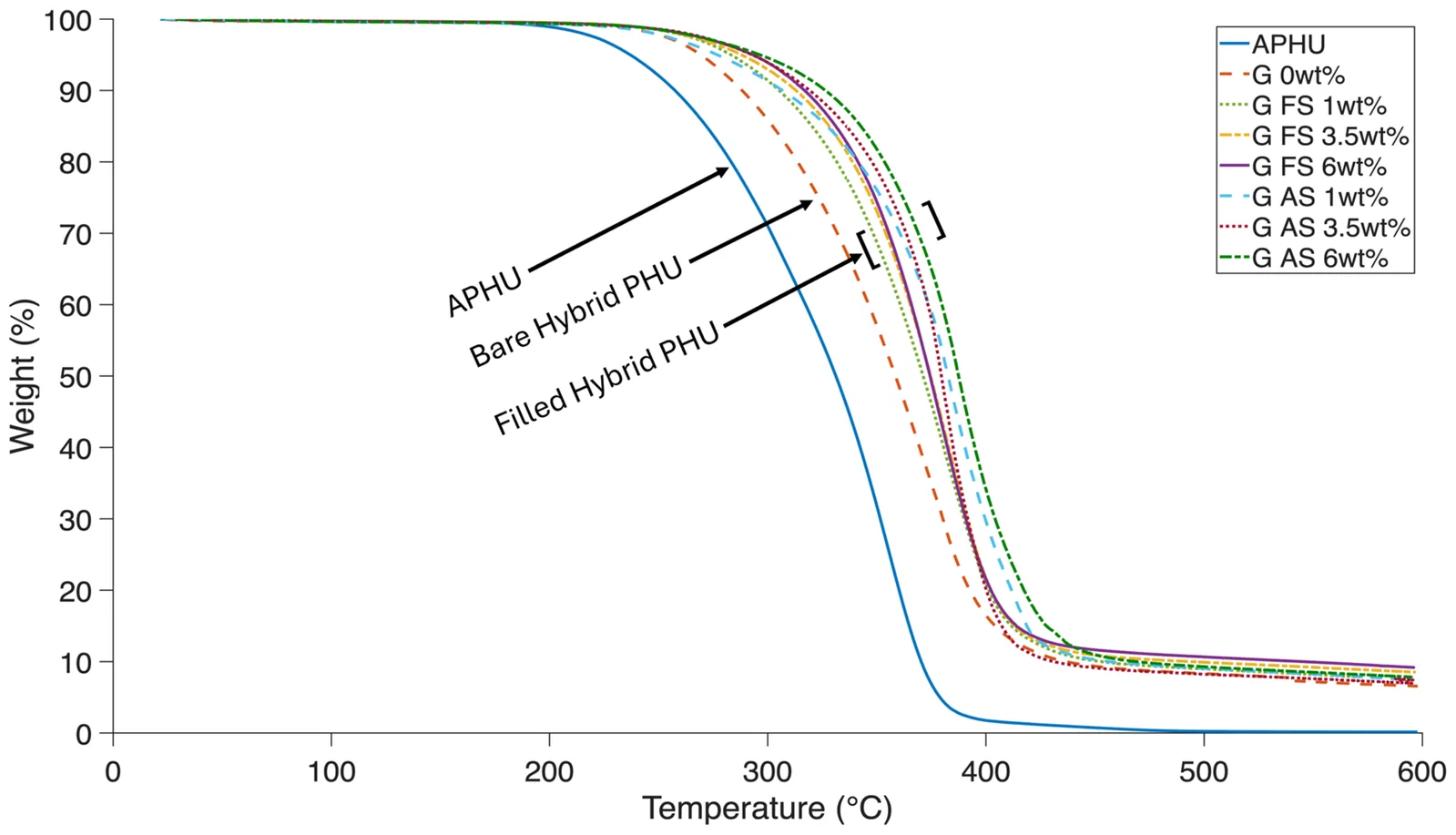
Thermogravimetric analysis results for GLYMO composites.

**Figure 11:**
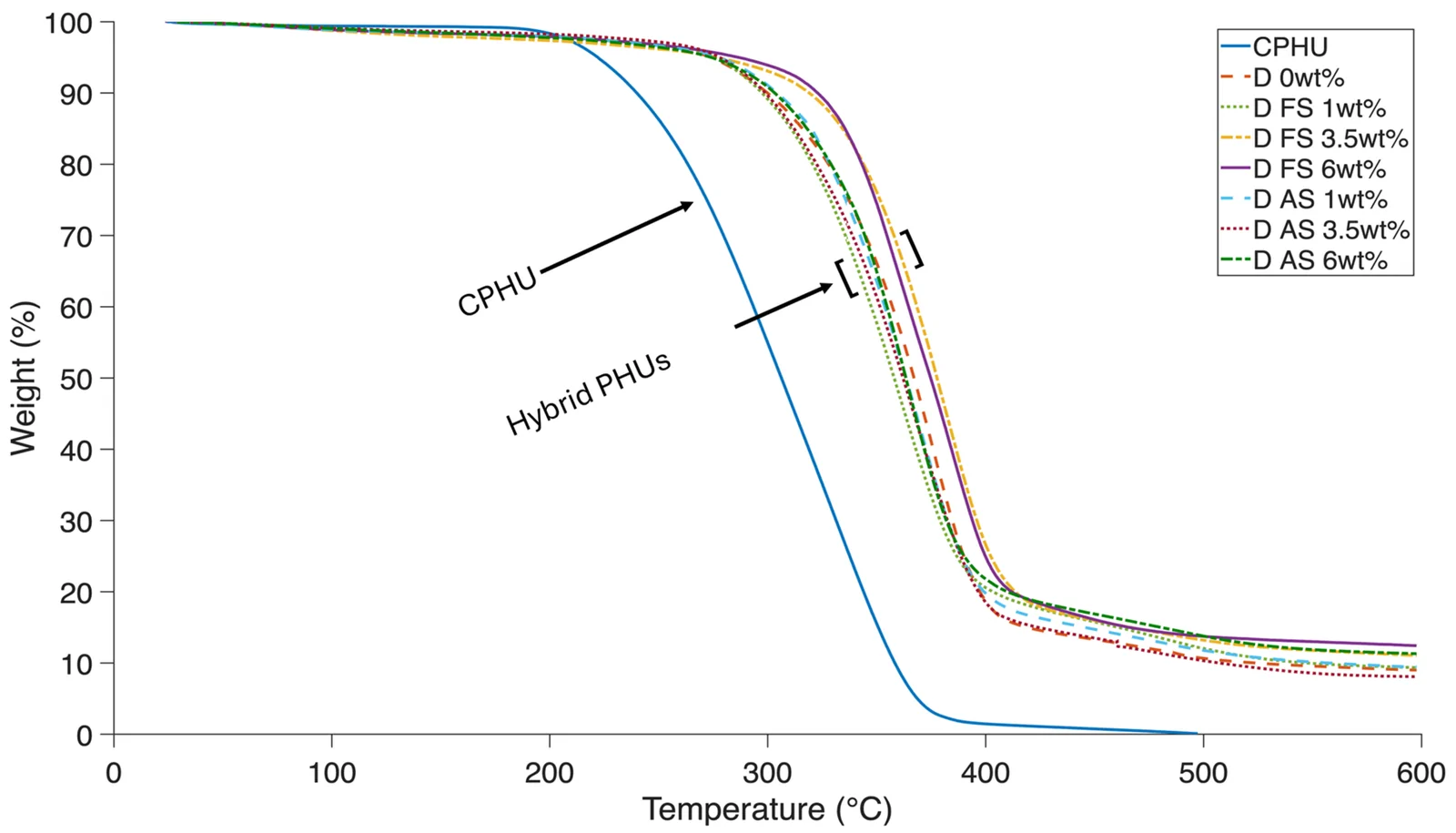
Thermogravimetric analysis results for DAMO composites.

DSC and TGA analyses were conducted on the hybrid PHUs to assess their thermal properties. Figure 10 and Figure 11 present the TGA curves for the two prepolymers and their corresponding composites.

The TGA data were analyzed using TRIOS software and are summarized alongside the DSC results in Table 4. A representative DSC curve is shown in Figure S18.

Analysis of the GLYMO composites’ degradation onset temperatures indicates that filler addition enhances thermal stability, as all reinforced GLYMO composites show higher onset temperatures than both APHU and the G_0wt% sample. Among the GLYMO composites, thermal stability generally improves with increasing filler content. In contrast, DAMO composites do not exhibit increased degradation onset with higher filler loadings. Although all DAMO composites are more thermally stable than CPHU, with T_d5%_ in agreement with Younes et al. [29], they remain less stable than GLYMO composites, likely due to their lower cross-linking density, which reduces thermal resistance. The DSC results show that the addition of the fillers for the GLYMO composites does not significantly impact the T_g_ of the composites relative to the bare sample nor are there any trends between the filler loading and type in both sets of composites. This could be due to the limited accuracy of the DSC instrument relative to the amount of filler present within the polymer matrices. Contrary to the increase in T_g_ when cross-linking the APHU, there is no significant change in T_g_ between the CPHU and the bare DAMO composite. This could be due to the incomplete curing discussed previously, which could leave the network with chain mobility like that of the PHU precursor.

#### 3.3.4. Nanoindentation

**Figure 12:**
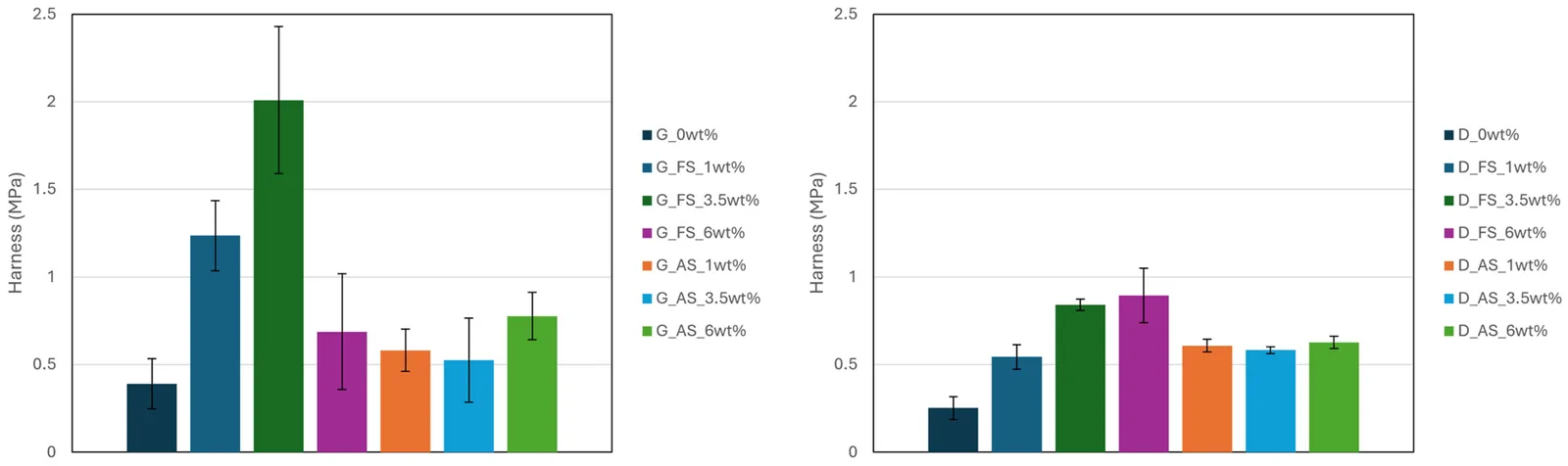
Hardness of GLYMO and DAMO composites (n = 3–5, standard deviation).

**Figure 13:**
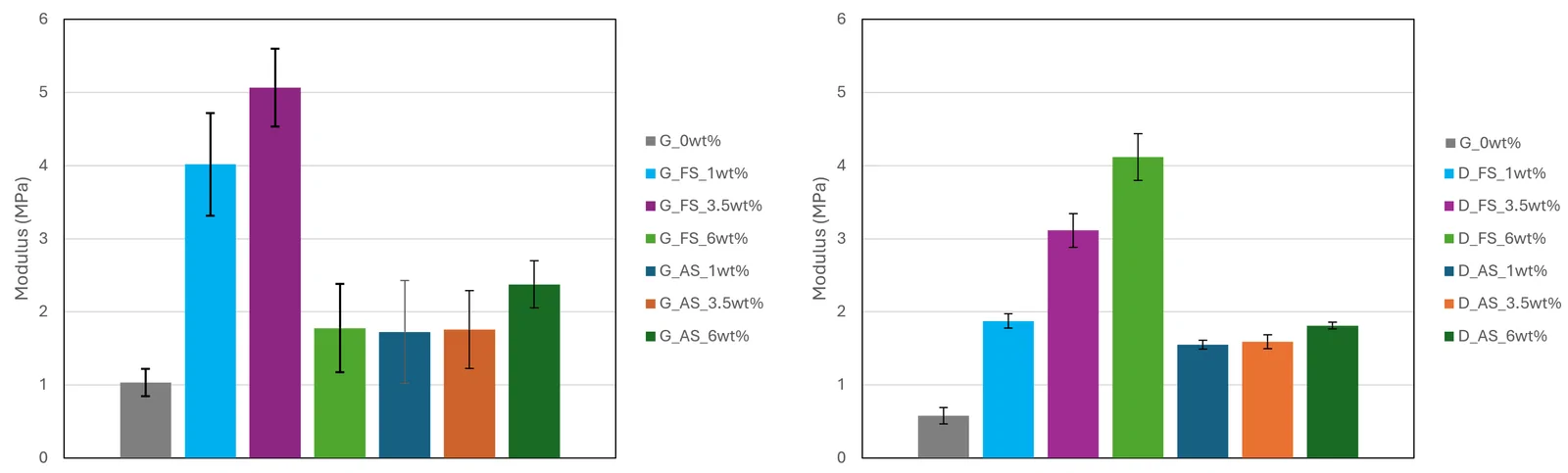
Modulus of GLYMO and DAMO composites (n = 3–5, standard deviation).

Nanoindentation tests were conducted to evaluate the hardness (HIT) and elastic modulus (EIT) of the composites, with the results shown in Figure 12 and Figure 13 revealing insight into the effect of filler on the PHU composites.

First, incorporating either filler enhances the mechanical properties of the hybrid PHUs. Second, all amino-functionalized silica composites exhibit lower hardness and modulus than their fumed silica counterparts. Third, multiple DAMO composites show inferior properties relative to GLYMO composites.

Initially, it was hypothesized that fumed silica would yield poorer mechanical properties due to its lack of covalent bonding with the polymer; however, the results show the opposite. Three potential explanations for this discrepancy are considered. One possibility is an incomplete reaction between GLYMO and the amino-functionalized silica. Excess GLYMO that did not react with the APHU may also not have reacted with the amino-functionalized silica, resulting in the physical entrapment of the filler and reduced performance. Given that the functionalized segments are softer than fumed silica (Figure S19), this would lower the overall mechanical properties. Low reactivity between Jeffamines and epoxies at ambient conditions may contribute to this incomplete reaction [30,31].

A second possibility is an insufficient molar concentration of amino groups on the functionalized silica relative to the excess GLYMO. As estimated in the supporting information, there was a molar excess of 17–104 mol GLYMO per mole of amine, meaning that there were insufficient functional filler particles to react with the GLYMO and significantly strengthen the matrix. This would explain the more modest improvements seen in hardness and modulus relative to fumed silica composites.

A third explanation is the formation of aggregates, documented in similar composites, which tend to improve properties relative to the neat polymer but degrade performance relative to a well-dispersed system [6,7,32,33]. Poor dispersion is plausible, as amino-functionalized silica visually required much longer ultrasonic dispersion times in ethyl acetate compared to fumed silica, 75 min and 15 min respectively. Furthermore, the ζ-potential of the filler (26.2 mV) is close to the 30 mV threshold for instability, indicating a high likelihood of aggregation [23]. SEM and TEM imaging confirmed this. Figure 12 and Figure 13 also show that composites containing 1 wt% and 3.5 wt% fumed silica exhibited the highest hardness and modulus, while the G_FS_6 wt% sample showed lower hardness and modulus, likely due to filler aggregation at higher loading. This is consistent with a report that higher filler contents can lead to agglomeration and diminished properties [6].

Finally, DAMO composites generally display poorer mechanical properties than GLYMO composites during nanoindentation. This agrees with the gel content and TGA results, which suggest a lower cross-link density in DAMO composites, yielding a less dense, more flexible, and mechanically weaker network. SEM and TEM analyses were conducted to further interpret the nanoindentation results and to assess the impact of dispersion on the composite structure and properties. Although the GLYMO composites with 1 wt% FS and 3.5 wt% FS displayed the highest indentation properties, their ability to maintain their properties overtime while exposed to different environmental factors should be investigated. Although outside the scope of this study, possible tests could include moisture resistance, UV aging, thermo-oxidative aging, and cyclic loading if used for industrial applications.

#### 3.3.5. Electron Microscopy

##### SEM Analysis

SEM imaging was performed to evaluate the silica nanoparticle dispersion in the reinforced PHU composites and to identify any aggregates. As a baseline, G_0wt% was imaged at various magnifications and is shown in Figure S4. After establishing the baseline, the G_FS_3.5wt% sample was analyzed since it had the highest hardness measured by nanoindentation. Inspection of the SEM images reveals no obvious aggregates. Overall, the SEM results, shown in Figure S5, suggest a uniform dispersion at the microscopic scale. However, given that fumed silica particle sizes range from 5 nm to 50 nm [34], TEM imaging was performed to confirm the filler’s presence and distribution within the matrix [15].

Following the analysis of the G_FS_3.5wt% sample, the G_AS_3.5wt% sample was examined to compare the AS dispersion. The SEM images, shown in Figure S6, reveal lamella-like structures unevenly dispersed throughout the sample. With increasing magnification, the lamella-like structures appear as aligned aggregates, prompting further TEM analysis of the G_AS_3.5wt% sample to better characterize these formations.

##### TEM Analysis

**Figure 14:**
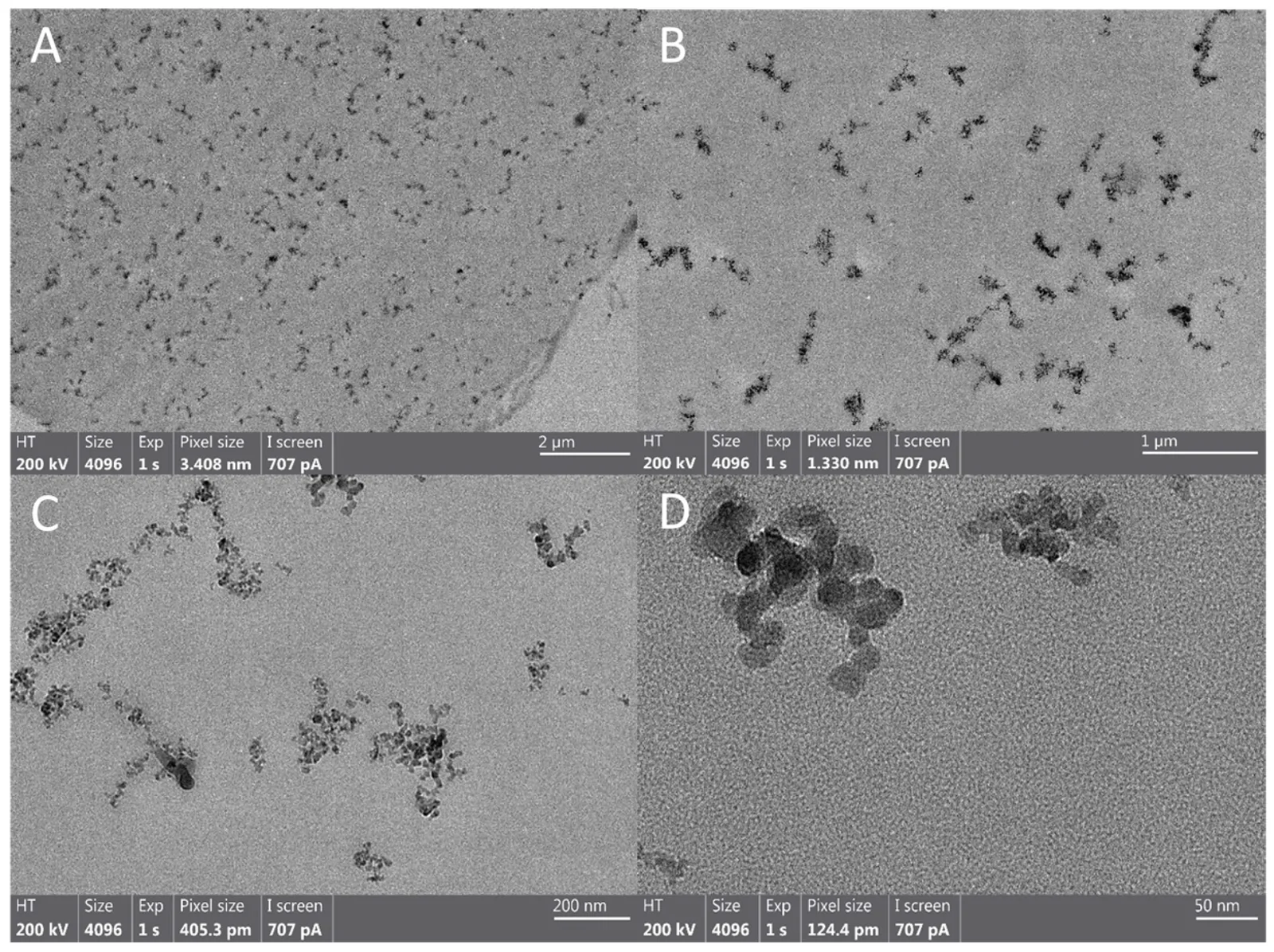
TEM of G_FS_3.5wt% sample at (**A**) 2 μm, (**B**) 1 μm, (**C**) 200 nm, (**D**) 50 nm. Reproduced with permission from Theo Bride, Incorporation of Inorganic and Organic Nanoparticles Within Moisture Curable Polyhydroxyurethane Matrices; published by McGill Libraries, 2025 [15].

The selected TEM images of the G_FS_3.5wt% sample are presented below in Figure 14.

Figure 14 shows no large aggregates, indicating that fumed silica is well-dispersed throughout the sample [15]. MATLAB (R2024A) analysis of multiple 1 μm scale images yielded an average dispersion fraction of 2.87% ± 0.058%, with a low standard deviation confirming uniform filler distribution. The methodology and code are detailed in the supporting information.

**Figure 15:**
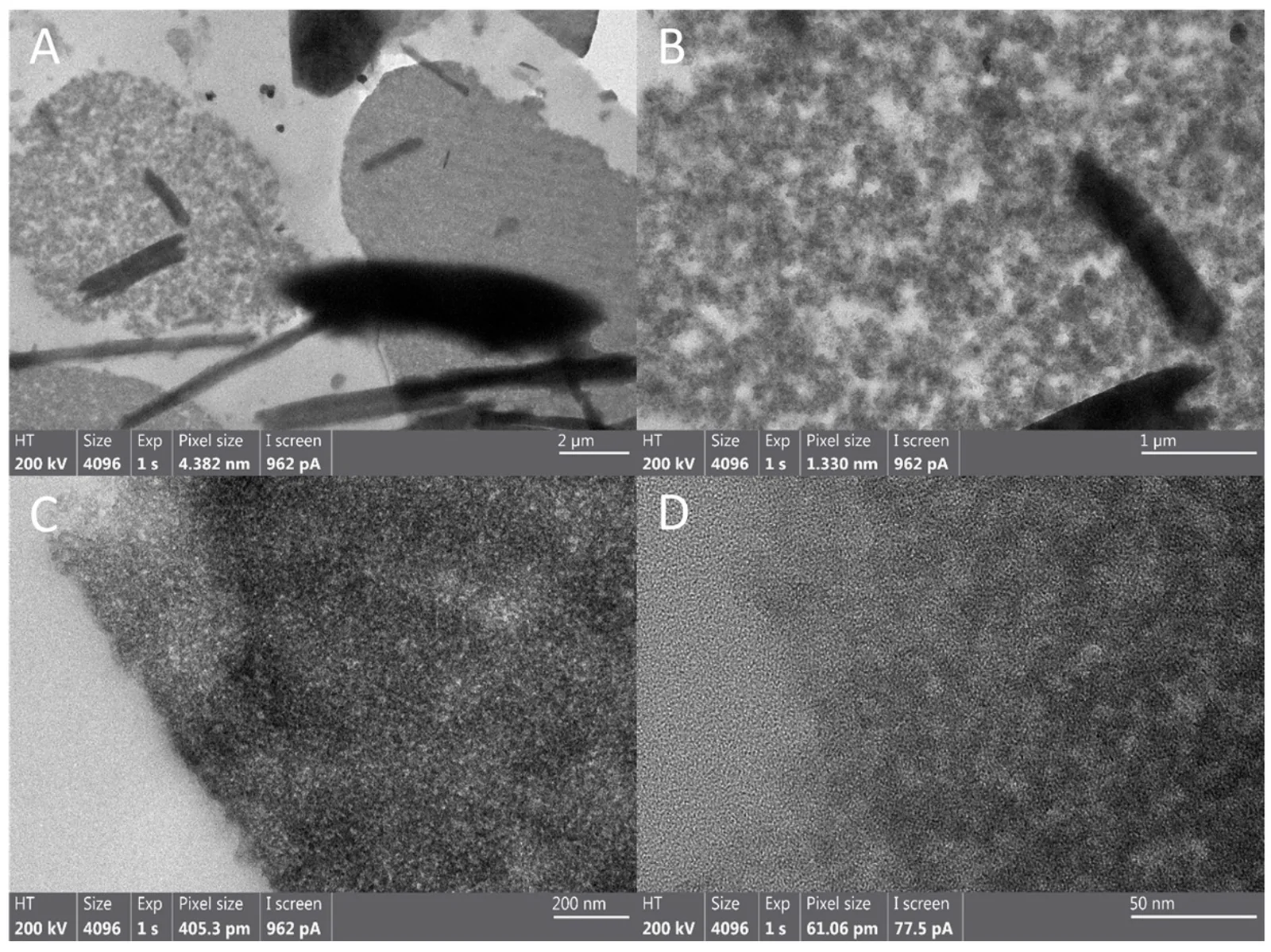
TEM of G_AS_3.5wt% sample at (**A**) 2 μm, (**B**) 1 μm, (**C**) 200 nm, (**D**) 50 nm. Reproduced with permission from Theo Bride, Incorporation of Inorganic and Organic Nanoparticles Within Moisture Curable Polyhydroxyurethane Matrices; published by McGill Libraries, 2025 [15].

TEM imaging was also conducted on the G_AS_3.5wt% sample to further examine the aggregates observed in SEM. Corresponding images at the same scale as Figure 14 are presented in Figure 15.

In contrast to the fumed silica TEM images, the TEM images from samples with amino-functional silica did not show distinct particles or small aggregates [15]. Instead, the lamella-like architecture observed in the SEM images was visible again and at 50 nm scale (Figure 15D), the amino-silica aggregates appear densely packed. The black visible in Figure 15A,B is debris formed when the sample was cut.

##### Particle Size and Elemental Analysis

**Figure 16:**
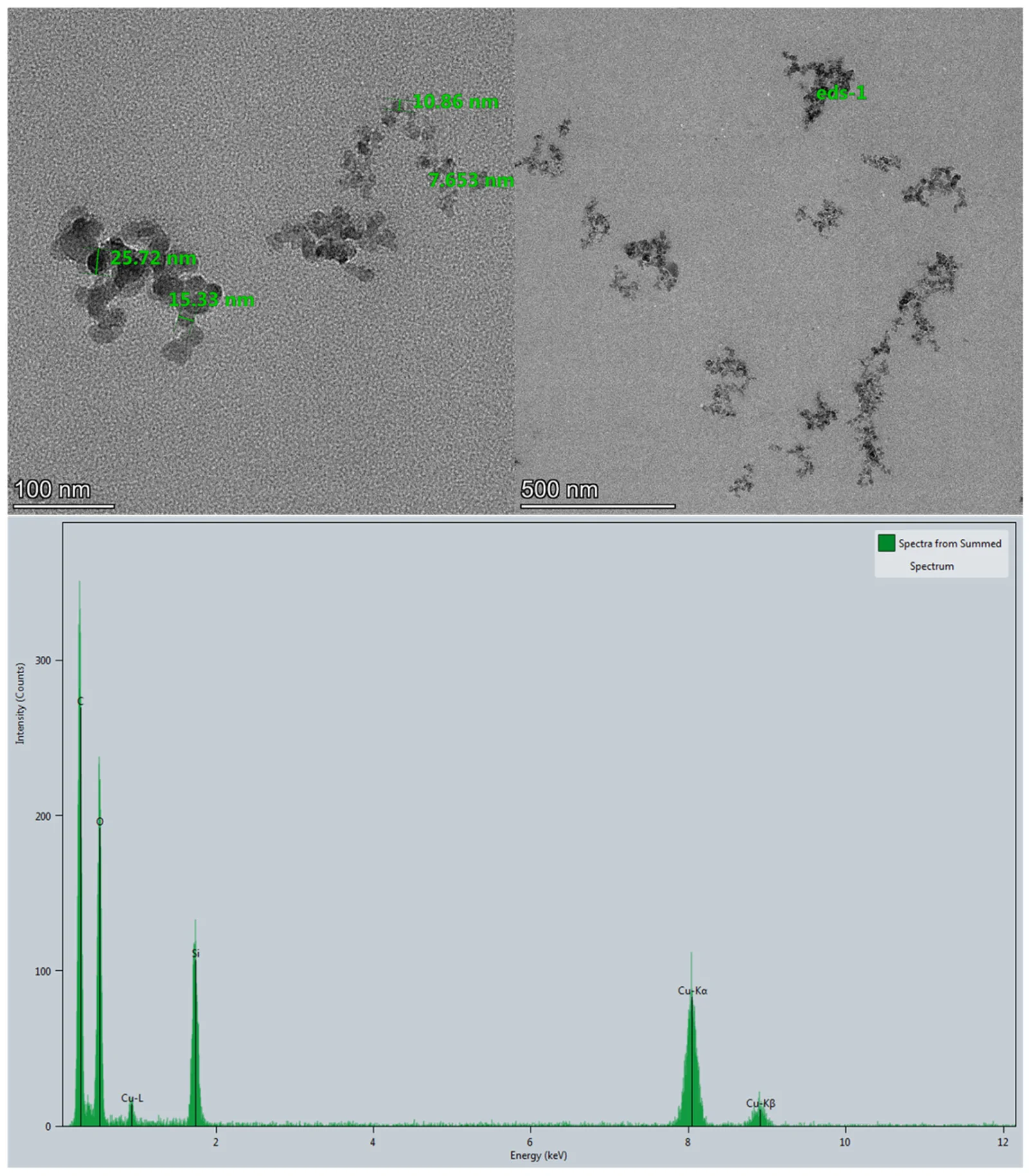
Particle size measurements and elemental analysis for G_FS_3.5wt%.

Particle size measurements were performed during the TEM analysis of the G_FS_3.5wt% sample to confirm that the observed features were individual particles rather than aggregates. Elemental analyses were also conducted to verify their composition as shown in Figure 16.

As depicted in Figure 16, measured particle sizes ranged from 7.5 nm to 26 nm, which is consistent with the nominal dimensions of fumed silica nanoparticles. This supports the conclusion that the fumed silica did not form large aggregates, or that any aggregates present exist only at the nanoscale. Elemental analysis revealed peaks for carbon, nitrogen (unlabeled peak between carbon and oxygen), oxygen, silicon, and copper (from the TEM grid), corresponding to the expected elements in the PHU samples.

#### 3.3.6. Tensile Testing

Tensile tests were conducted to determine the Young’s modulus, ultimate strain, and ultimate stress of the samples. Tests were limited to the G_0wt% and G_FS_3.5wt% composites, as nanoindentation indicated that the latter had the most promising mechanical properties. During the tensile tests, the G_0wt% specimens failed too rapidly for data collection, whereas meaningful data were obtained for the G_FS_3.5wt% specimens. These results support the conclusion that filler incorporation enhances composite mechanical performance. The stress–strain curves are shown in Figure S20, and the key parameters are summarized in Table 5.

Compared to other reinforced PHUs reported in the literature, the tested samples exhibited relatively modest improvements in mechanical properties [8,19]. In comparison to the findings of MacInnis et al., who reported a Young’s modulus ranging from 1.7 to 5.9 MPa, an ultimate stress between 0.55 and 1.32 MPa, and an ultimate strain of 17–46%, the weaker tensile properties identified in this study can be primarily attributed to two factors. Firstly, a considerably higher cross-linking density, resulting from the increased availability of pendant hydroxyl groups which were reacted with 3-isocyanatopropyl(trimethoxy) silane, significantly contributes to the superior tensile properties observed in their work. Secondly, the use of amino-functionalized POSS as a filler yields a filler chemically bound to the polymer network, contrasting the physical bonding exhibited by fumed silica, thus facilitating a more effective reinforcement of the polymer sample. Similarly, when compared to the findings of Blattman and Mülhaupt, who reported a Young’s modulus ranging from 25 to 4000 MPa, an ultimate stress between 8.4 and 87 MPa, and an ultimate strain of 0.4–32%, the inferior tensile properties observed in this study can be primarily attributed to two factors. The cyclic carbonate-functionalized POSS was also chemically bound, and the relative weight percent of POSS cross-linker/filler was more prevalent in their system. Nevertheless, the results demonstrated that the incorporation of fumed silica in our system still enhanced the composite’s properties relative to the unfilled PHU. This study emphasized the scalability and industrial implementation of a reinforced PHU system by mimicking existing conventional polyurethanes thermoset formulations and utilizing a commercially available diamine and fumed silica fillers. As sol–gel processes are common practice within different polymer subfields, the scalability and implementation of the synthesis procedure could be achieved, with the only concern being to add a solvent capture system to minimize environmental impacts and the use of the ethyl acetate solvent through post-evaporation recovery.

## 4. Conclusions

This study investigated the effects of silica-based fillers on the mechanical properties of a moisture curing matrix. A polyhydroxyurethane prepolymer was synthesized in bulk using Jeffamine D-2000 and DGC. It was subsequently mixed with a moisture curing agent (GLYMO or DAMO), a solvent (ethyl acetate), a catalyst (triethylamine), and a filler (fumed silica (FS), amino-silica (AS), or none). The mixture was placed in a silicone mold and cured under ambient conditions. Samples were characterized by swelling tests, TGA, DSC, FTIR, SEM, and TEM. TGA indicated that increasing filler content enhanced the thermal stability of GLYMO composites. Specifically, the 6 wt% AS sample exhibited a 5 wt% degradation temperature of 296 °C, closely followed by the 6 wt% FS and 3.5 wt% AS samples (~293 °C). Gel content tests showed ≥60% cross-linking for all formulations, with GLYMO composites yielding higher cross-link density than DAMO. SEM and TEM revealed well-dispersed fumed silica but significant aggregation of AS. These aggregates likely contributed to the poor performance of PHUs with amino-functionalized silica in the nanoindentation tests. Indeed, the G_FS_3.5wt% sample had the highest hardness, 2.0 MPa, and the highest modulus, 5.1 MPa. Tensile tests supported these findings, as the addition of a filler increased the Young’s modulus, σ_u_, and ε_u_. These were 0.0280 MPa, 0.118 MPa, and 13.2% respectively, all of which were unobtainable for the benchmark PHU as it was mechanically too weak. Overall, both fillers enhanced the mechanical properties of the hybrid PHU, though poor dispersion limited the amino-functionalized silica’s effectiveness. Future work should access the effectiveness of each filler using alternative dicarbonates and diamines and should increase the weight percent of amino-functionalized silica while reducing the mol percent of the moisture curing agents DAMO and GLYMO. Additionally, future work should investigate the long-term stability of the materials synthesized through tests such as thermo-oxidative aging, moisture resistance, and UV aging.

## Figures and Tables

**Table 1:** Nomenclature of composites analyzed.

Sample Name	PHU	Moisture Curing Agent	Filler Used	Filler wt%
G_0wt%	APHU	GLYMO	None	0
G_FS_1wt%	APHU	GLYMO	Fumed silica	1
G_FS_3.5wt%	APHU	GLYMO	Fumed silica	3.5
G_FS_6wt%	APHU	GLYMO	Fumed silica	6
G_AS_1wt%	APHU	GLYMO	Amino modified silica	1
G_AS_3.5wt%	APHU	GLYMO	Amino modified silica	3.5
G_AS_6wt%	APHU	GLYMO	Amino modified silica	6
D_0wt%	CPHU	DAMO	None	0
D_FS_1wt%	CPHU	DAMO	Fumed silica	1
D_FS_3.5wt%	CPHU	DAMO	Fumed silica	3.5
D_FS_6wt%	CPHU	DAMO	Fumed silica	6
D_AS_1wt%	CPHU	DAMO	Amino modified silica	1
D_AS_3.5wt%	CPHU	DAMO	Amino modified silica	3.5
D_AS_6wt%	CPHU	DAMO	Amino modified silica	6

**Table 2:** Gel content tests with water and toluene.

Sample	Gel Content in Water (%)	Gel Content in Toluene (%)
G_0wt%	98.5 (±0.5)	86.5 (±0.4)
G_FS_1wt%	93.7 (±1.1)	81.0 (±3.1)
G_FS_3.5wt%	97.0 (±0.5)	85.1 (±5.5)
G_FS_6wt%	95.1 (±0.0)	77.2 (±0.5)
G_AS_1wt%	98.1 (±0.2)	77.9 (±0.3)
G_AS_3.5wt%	97.7 (±0.5)	80.5 (±2.7)
G_AS_6wt%	97.5 (±0.4)	86.4 (±0.8)
D_0wt%	94.1 (±1.0)	69.2 (±0.7)
D_FS_1wt%	90.4 (±2.5)	69.8 (±0.4)
D_FS_3.5wt%	89.6 (±1.2)	65.3 (±0.1)
D_FS_6wt%	90.7 (±0.7)	63.7 (±1.7)
D_AS_1wt%	92.9 (±0.5)	66.0 (±2.4)
D_AS_3.5wt%	92.5 (±0.4)	63.8 (±2.1)
D_AS_6wt%	87.0 (±1.6)	66.7 (±0.7)

**Table 3:** Tabulated version swelling tests.

Sample	Swelling Index H_2_O (%)	Swelling Index Toluene (%)
G_0wt%	5.2 (±0.2)	166.9 (±11.6)
G_FS_1wt%	11.3 (±1.2)	222.4 (±5.3)
G_FS_3.5wt%	5.1 (±0.1)	180.0 (±6.6)
G_FS_6wt%	4.5 (±0.4)	231.2 (±17.2)
G_AS_1wt%	5.5 (±0.3)	194.5 (±4.9)
G_AS_3.5wt%	6.0 (±0.6)	192.5 (±5.8)
G_AS_6wt%	6.3 (±0.5)	179.7 (±4.6)
D_0wt%	67.7 (±1.1)	167.3 (±2.7)
D_FS_1wt%	46.0 (±5.7)	151.6 (±0.8)
D_FS_3.5wt%	48.6 (±1.7)	108.1 (±1.3)
D_FS_6wt%	51.4 (±1.7)	90.1 (±2.4)
D_AS_1wt%	74.9 (±5.9)	142.7 (±6.5)
D_AS_3.5wt%	63.6 (±4.4)	135.8 (±8.1)
D_AS_6wt%	49.9 (±0.6)	132.6 (±2.6)

**Table 4:** This table shows the 5% degradation temperature and T_g_s of the prepolymers and composites.

Name	T_onset_ (5%) (°C)	T_g_ (°C)
APHU	237	−64
G_0wt%	269	−59
G_FS_1wt%	283	−59
G_FS_3.5wt%	288	−59
G_FS_6wt%	293	−59
G_AS_1wt%	277	−59
G_AS_3.5wt%	293	−60
G_AS_6wt%	296	−60
CPHU	210	−59
D_0wt%	256	−60
D_FS_1wt%	257	−59
D_FS_3.5wt%	244	−60
D_FS_6wt%	268	−60
D_AS_1wt%	265	−60
D_AS_3.5wt%	263	−60
D_AS_6wt%	245	−60

**Table 5:** Tensile measurements of G_FS_3.5wt% sample.

	Young’s Modulus (MPa)	Ultimate Stress (MPa)	Ultimate Strain (%)
Tensile bar 1	0.023	0.079	11
Tensile bar 2	0.027	0.100	13
Tensile bar 3	0.034	0.170	16
Average	0.028	0.120	13
Standard deviation	0.005	0.036	2.5

## Data Availability

The data supporting this article have been included as part of the ESI. Data for this article, including all raw data tabulated in Microsoft Excel or CSV format for DSC, TGA, GPC, Rheology, FTIR and ^1^H NMR measurements are available at McGill University Dataverse [https://borealisdata.ca/dataverse/mcgill] at https://doi.org/10.5683/SP4/Q2UOD7 (accessed on 19 August 2026).

## Supplementary Materials

The following supporting information can be downloaded at https://www.mdpi.com/article/10.3390/polym18172061/s1. Figure S1: Integration of benzophenone proton peak; Figure S2: Integration of PHU peak of interest (protons in NH2 functional groups); Figure S3: Confirmation of peak disappearance after use of phenyl isocyanate to quench amines; Figure S4: SEM of G_0wt% sample a magnification of (A) 250X, (B) 1000X, (C) 5000X, (D) 20,000X (these pictures serve as baselines for other SEM images); Figure S5: SEM of G_FS_3.5wt% sample at a magnification of (A) 250X, (B) 1000X, (C) 5000X, (D) 20,000X The large white debris are likely sample fragments produced during cutting. Notably, the copper grid is visible beneath the sample in B, C, and D due to its minimal thickness; Figure S6: SEM of G_AS_3.5wt% sample at a magnification of (A) 250X, (B) 1000X, (C) 5000X, (D) 20,000X; Figure S7: Color inversion of TEM picture using a threshold of 97 on the grayscale; Figure S8: Inverted TEM image with a filter of 6; Figure S9: TEM image with threshold of 50 and no filter; Figure S10: TEM image with threshold of 98 and no filter; Figure S11: TEM image with threshold of 97 and filter 10; Figure S12: TEM image with threshold of 97 and filter 6; Figure S13: MATLAB code for TEM image analysis; Figure S14: Contact angle vs. time; Figure S15: Viscosity of amine terminated PHU Prepolymer for different shear rates; Figure S16: Visual depiction of fillers; Figure S17: GPC trace of pure Jeffamine D-2000 (Mn of 3600, Mw of 3800, and Đ of 1.054); Figure S18: Silane coupling agent curing mechanism; Figure S19: 2nd heating ramp of APHU for DSC analysis; Figure S20: Graph of the 3 tensile tests. Table S1: Contact angle measurements; Table S2: Default values for NMR test parameters.

## Abbreviations

The following abbreviations are used in this manuscript:

PHU	Polyhydroxyurethanes
GLYMO	3-(2,3-epoxypropoxy)-propyl]-trimethoxysilane
PU	Polyurethanes
NIPU	Non-isocyanate Polyurethanes
MDI	Methylene Diisocyanate
TDI	Toluene Diisocyanate
CMR	Carcinogenic, Mutagenic, and Reprotoxic
FS	Fumed Silica
AS	Amino-functionalized Silica
POSS	Polyhedral Oligomeric Silsequioxanes
DAMO	N-(2-aminoethyl)-3-aminopropyltrimethoxysilane
DGC	Diglycerol Carbonate
APHU	Amine-terminated Polyhydroxyurethanes
CPHU	Cyclic Carbonate-terminated Polyhydroxyurethanes
GC	Gel Content
DSC	Differential Scanning Calorimetry
SEM	Scattering Electron Microscopy
TEM	Transmission Electron Microscopy
FTIR	Fourier Transfer Infrared Spectroscopy
NMR	Nuclear Magnetic Resonance Spectroscopy
THF	Tetrahydrofuran
RO	Reverse Osmosis
TGA	Thermogravimetric Analysis
GPC	Gel Permeation Chromatography
*M*_w_	Weight Average Molecular Weight
*M*_*n*_	Number Average Molecular Weight
