# Supplementary material for: Moisture-Curable Poly(hydroxyurethanes) Composites Reinforced with Silica Nanofillers
Source: Polymers (Basel). 2026 Aug 25;18(17):2061. doi: 10.3390/polym18172061 (PMC13568025; doi:10.3390/polym18172061)
Supplement: Supplementary file 1 [file polymers-18-02061-s001.zip › polymers-4475471-supplementary.pdf]

Supporting Information

for

Moisture Curable PolyHydroxyUrethanes composite /reinforced with Nanofillers

Theo Bride, Eline Laurent, Jad Raad, Milan Marić *

Department of Chemical Engineering, McGill University, Montreal, Quebec, Canada, H3A 0C5

*Email: milan.maric@mcgill.ca

Table of Contents

S1. NMR Molecular Weight Calculation 3

S2. Calculation of GLYMO to Amine Mol Ratio 4

S3. SEM Characterization of Polymers 5

S4. TEM Dispersion Analysis 7

S5. Water Contact Angle Measurements 12

S6. Viscosity Measurement of Amine terminated PHU 14

S7. Additional Tables and Figures 14

Table of Figures

Figure S1: Integration of benzophenone proton peak 3

Figure S2: Integration of PHU peak of interest (protons in NH_2 functional groups) 4

Figure S3: Confirmation of peak disappearance after use of phenyl isocyanate to quench amines 4

Figure S4: SEM of G_0wt% sample a magnification of A) 250X, B) 1000X, C) 5000X, D) 20000X (these pictures serve as baselines for other SEM images) 5

Figure S5: SEM of G_FS_3.5 wt% sample at a magnification of A) 250X, B) 1000X, C) 5000X, D) 20000X The large white debris are likely sample fragments produced during cutting. Notably, the copper grid is visible beneath the sample in B, C, and D due to its minimal thickness. 6
Figure S6: SEM of G_AS_3.5wt% sample at a magnification of A) 250X, B) 1000X, C) 5000X, D) 20000X 7
Figure S7: Color inversion of TEM picture using a threshold of 97 on the grayscale 8
Figure S8: Inverted TEM image with a filter of 6 8
Figure S9: TEM image with threshold of 50 and no filter........ 9
Figure S10: TEM image with threshold of 98 and no filter........ 10
Figure S11: TEM image with threshold of 97 and filter 10........ 10
Figure S12: TEM image with threshold of 97 and filter 6........ 11
Figure S13: MATLAB code for TEM image analysis........ 12
Figure S14: Contact angle vs time 13
Figure S15: Viscosity of amine terminated PHU Prepolymer for different shear rates 14
Figure S16: Visual depiction of fillers........ 16
Figure S17: GPC trace of pure Jeffamine D-2000 (M_n of 3600, M_w of 3800, and Đ of 1.054) ... 15
Figure S18: Silane coupling agent curing mechanism........ 15
Figure S19: 2nd heating ramp of APHU for DSC analysis........ 16
Figure S20: Graph of the 3 tensile tests........ 17

Table of Tables

Table S1: Contact angle measurements 13
Table S2: Default values for NMR test parameters........ 14

S1. NMR Molecular Weight Calculation

For the project at hand, benzophenone served as the internal standard. The signal integration was performed within the 7.3-8 ppm range, where each molecule of benzophenone contains 10 protons. Due to this, the integral is assigned a value of 10. The polymer peak of interest (protons on NH_2 functional groups and cyclic carbonate) was integrated, ensuring proper identification through literature or other means, such as confirming the location of the NH_2 peak using phenyl isocyanate (which reacts with NH_2 groups). Upon integration, values were input into the following equation to calculate average molecular weight:

$$\frac{Benzo\ peak\ integration * \#\ of\ protons\ PHU\ peak * Mass\ of\ PHU * Molar\ mass\ of\ Benzo}{PHU\ peak\ integration * \#\ of\ protons\ of\ Benzo * Mass\ of\ Benzo}$$

This equation gives the equivalent weight per functional group (EWFG) which is then multiplied by the estimated number of functional groups (ENFG) to give the molecular weight as follows:

$$Mw = ENFG * EWFG$$

An example of an NMR graph is given below:

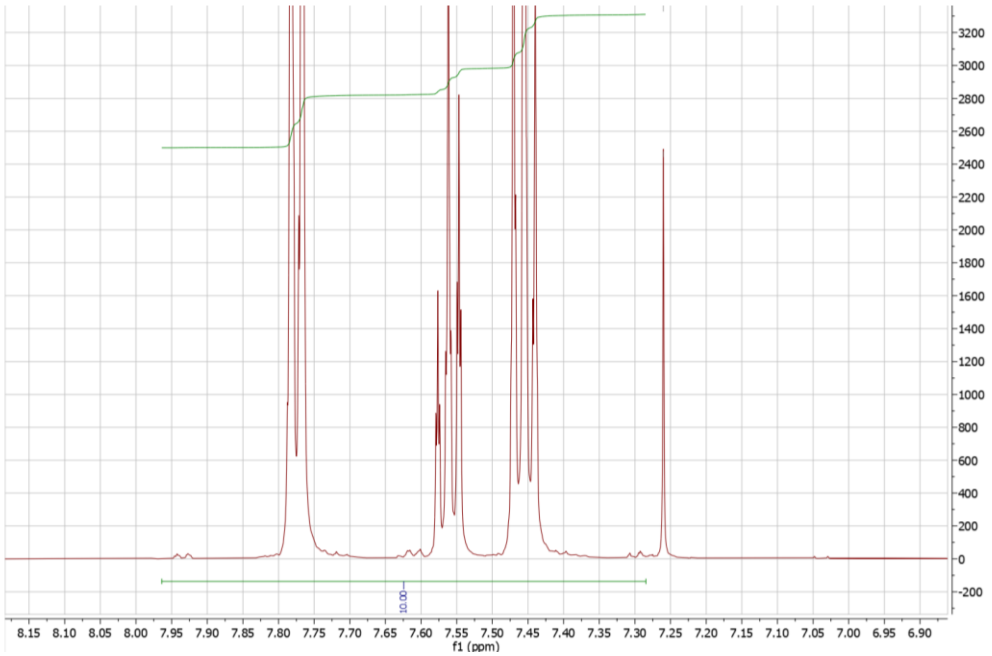

Figure S1: Integration of benzophenone proton peak

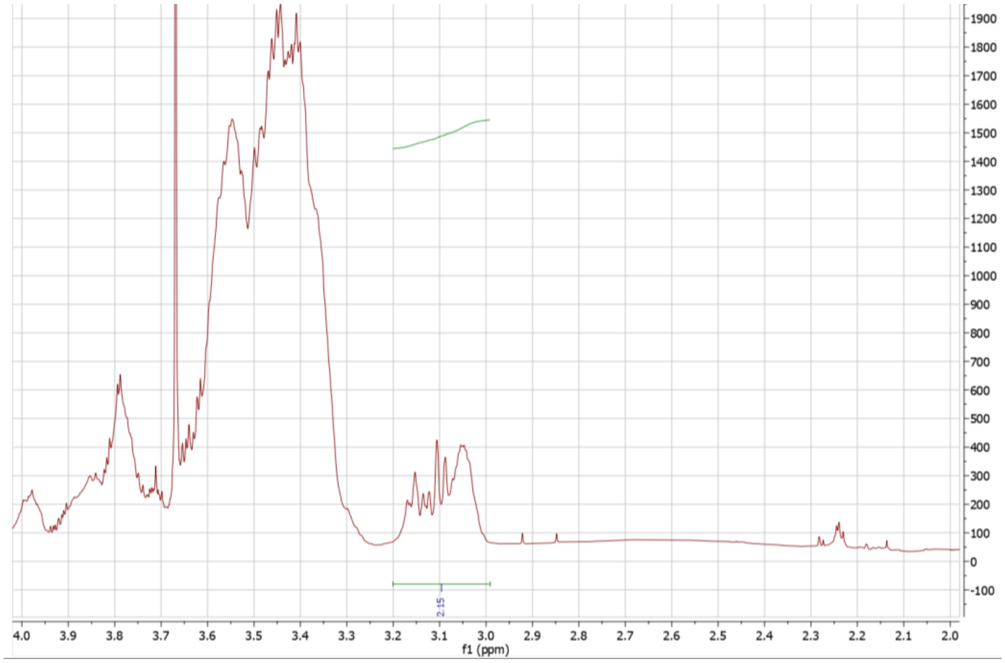

Figure S2: Integration of PHU peak of interest (protons in NH_2 functional groups)

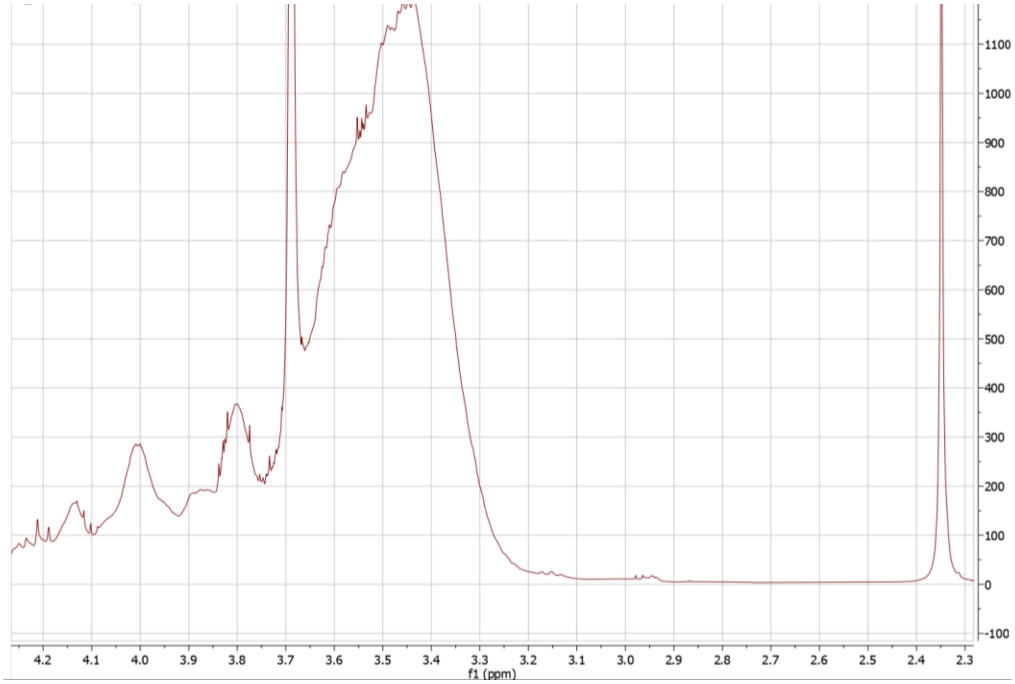

Figure S3: Confirmation of peak disappearance after use of phenyl isocyanate to quench amines

S2. Calculation of GLYMO to Amine Mol Ratio

$$Mols\ of\ GLYMO = \frac{0.35g}{\frac{236.34g}{mol}} = 0.00148\ mols\ of\ GLYMO$$

$$Mols\ of\ GlYMO\ if\ all\ PHU\ amines\ reacted = 0.00148\ mol - 0.00074\ mol = 0.0074\ mol$$

$$Mols\ of\ amines\ for\ 1wt\%\ of\ Amino\ silica = \frac{mass\ of\ PHU * weight\ percent\ of\ filler * weight\ percent\ of\ nitrogen\ infiller}{atomic\ weight\ of\ nitrogen}$$

$$Mols\ of\ amines\ for\ 1\ wt\%\ of\ Amino\ silica = 2g\ * \frac{1}{100} * \frac{0.5}{100} * \frac{1mol\ nitrogen}{14g\ nitrogen} = 0.00000714mols\ nitrogen$$

$$Ratio\ of\ GLYMO\ to\ filler\ amines = \frac{0.0074mols\ GYMO}{0.00000714mols\ amine} = 103.7$$

S3. SEM Characterization of Polymers

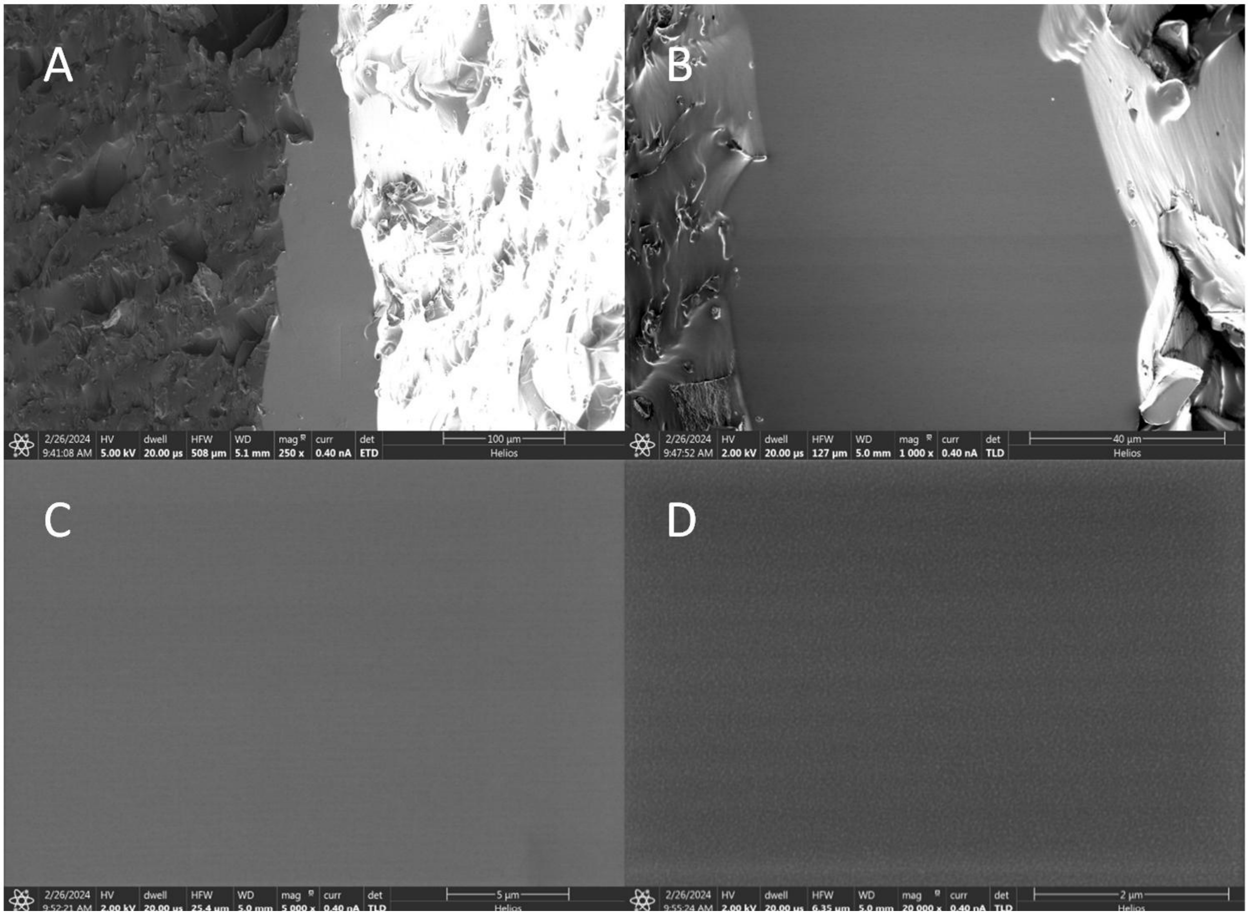

Figure S4: SEM of G_0wt% sample a magnification of A) 250X, B) 1000X, C) 5000X, D) 20000X (these pictures serve as baselines for other SEM images)

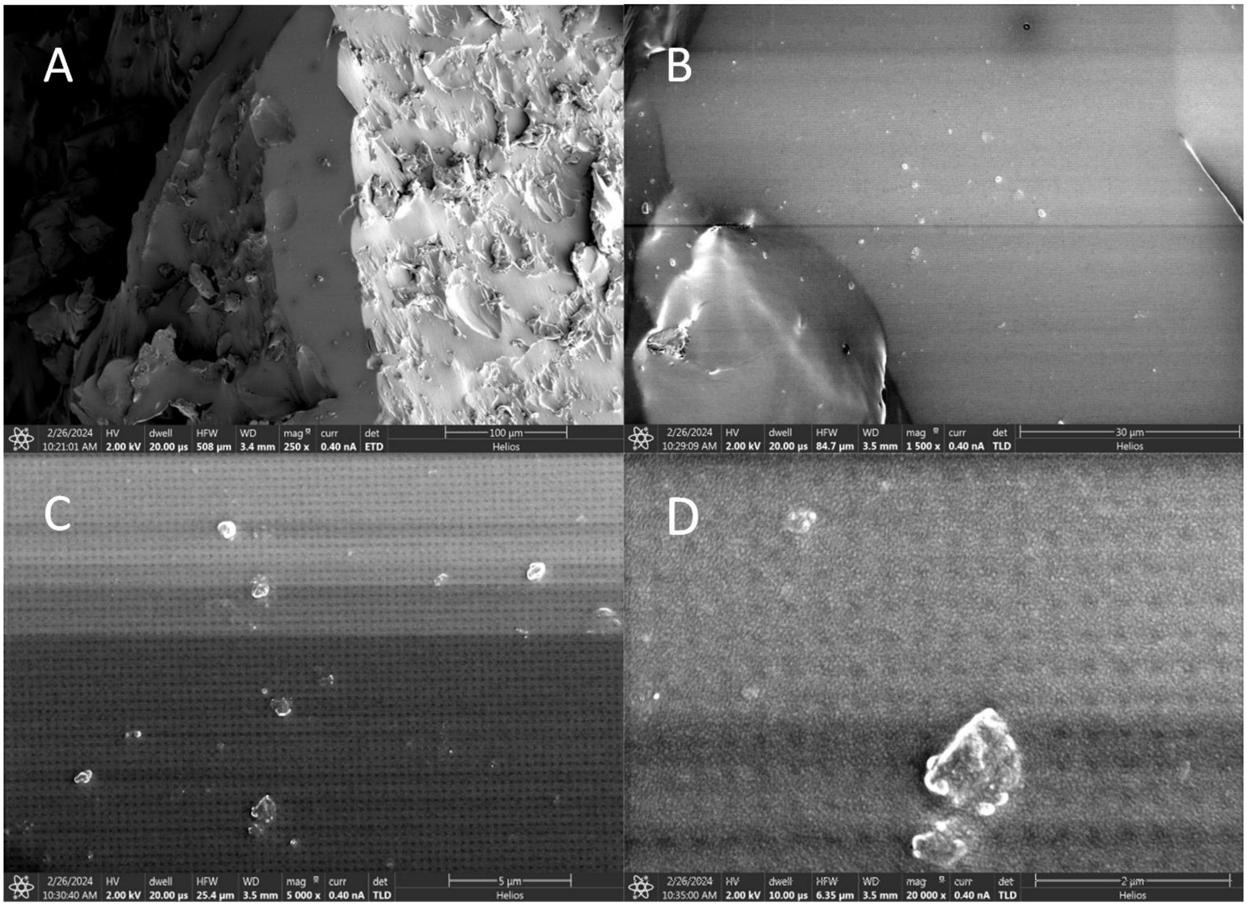

Figure S5: SEM of G_FS_3.5 wt% sample at a magnification of A) 250X, B) 1000X, C) 5000X, D) 20000X The large white debris are likely sample fragments produced during cutting. Notably, the copper grid is visible beneath the sample in B, C, and D due to its minimal thickness.

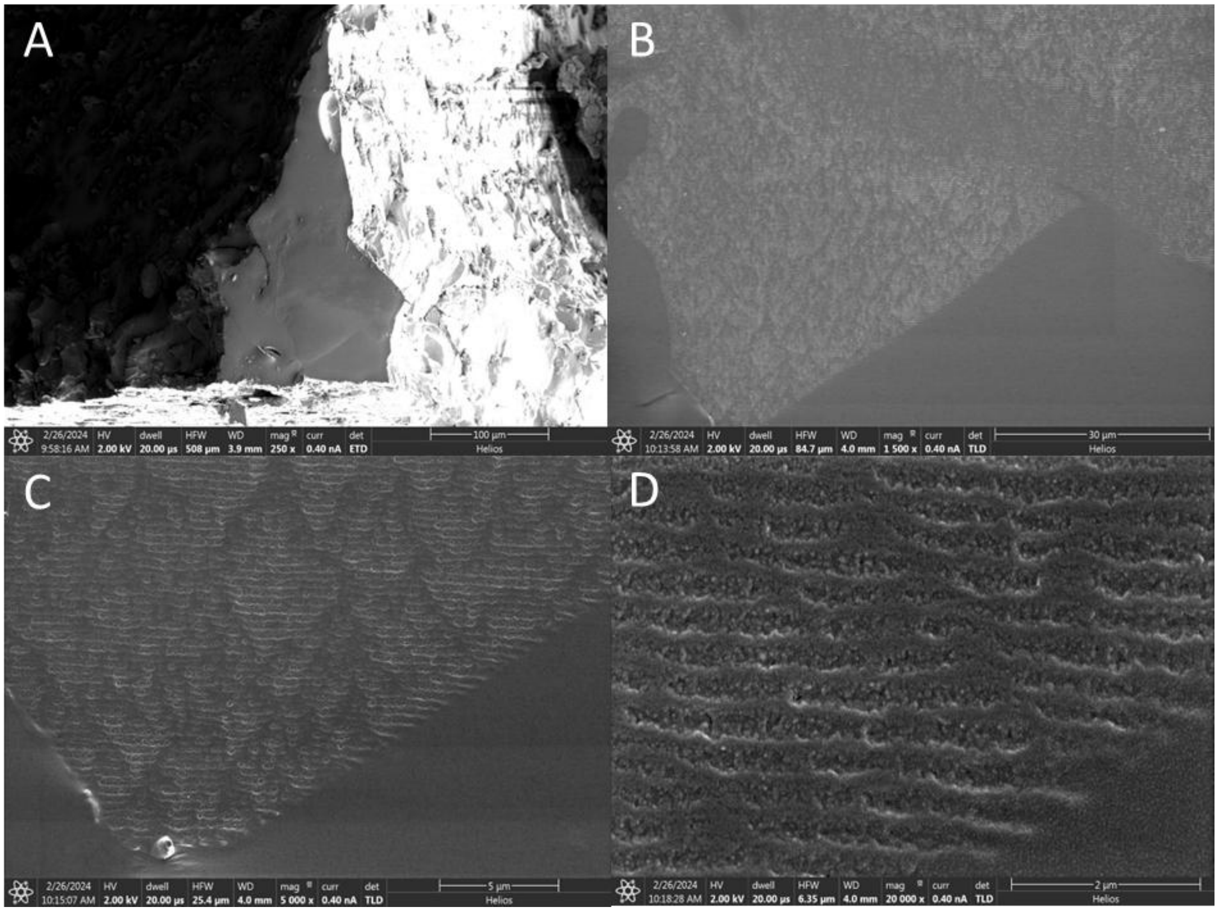

Figure S6: SEM of G_AS_3.5wt% sample at a magnification of A) 250X, B) 1000X, C) 5000X, D) 20000X

S4. TEM Dispersion Analysis

The objective of the MATLAB script was to ascertain the number of black pixels within a TEM image. First, color inversion was achieved through grayscale transformation and setting an appropriate threshold. For instance, the following code snippet demonstrates inversion using a threshold value of 97:

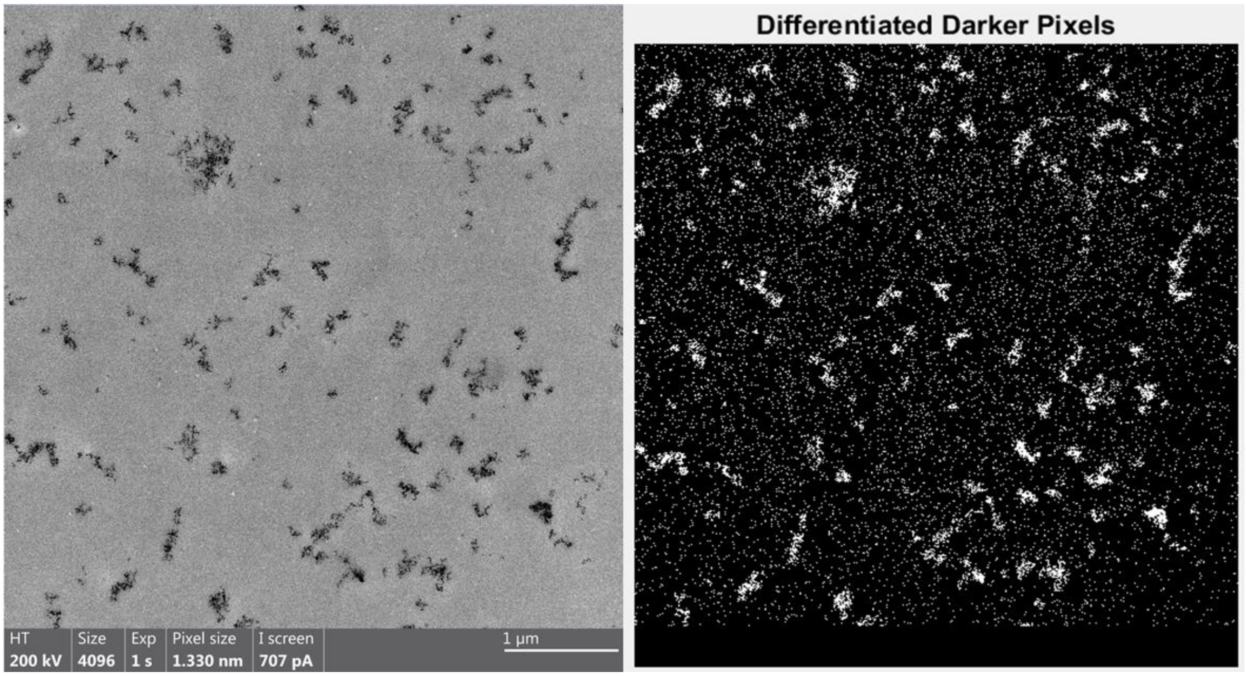

Figure S7: Color inversion of TEM picture using a threshold of 97 on the grayscale

A filter was then added to remove all the small pixels between the particles. An example is shown below:

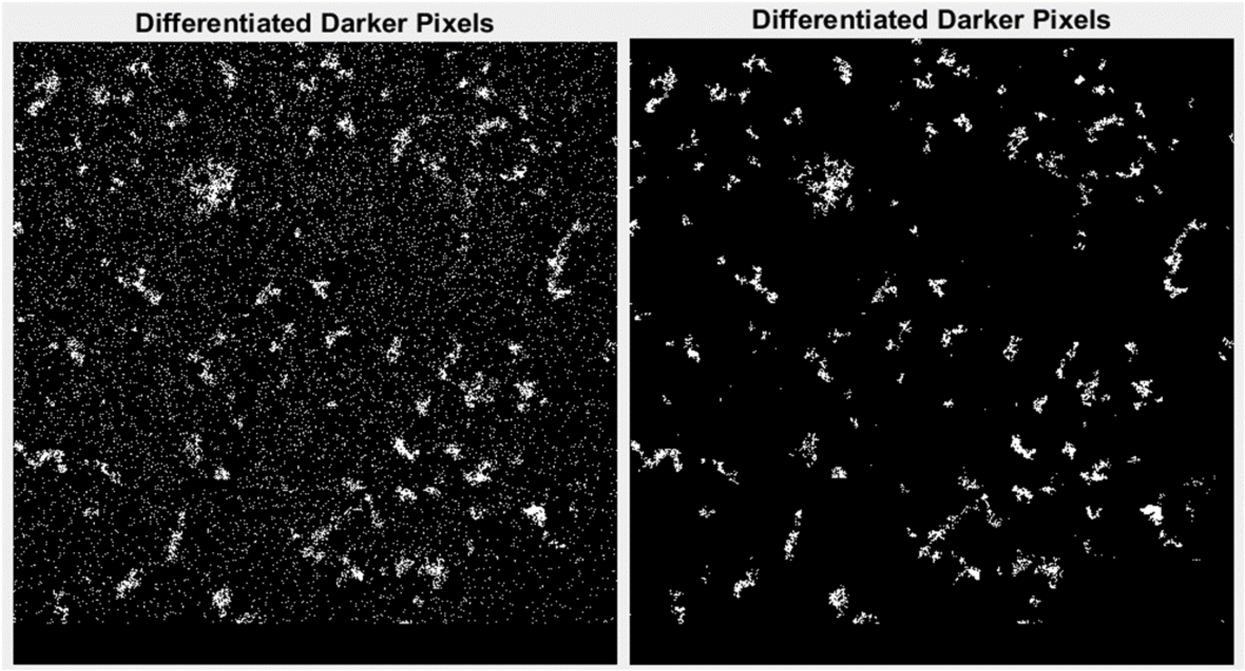

Figure S8: Inverted TEM image with a filter of 6

From here on, different thresholds were tested and then a filter was added. I zoomed in to one of the filler particles to get a good idea of the accuracy of the values. Some examples are shown below:

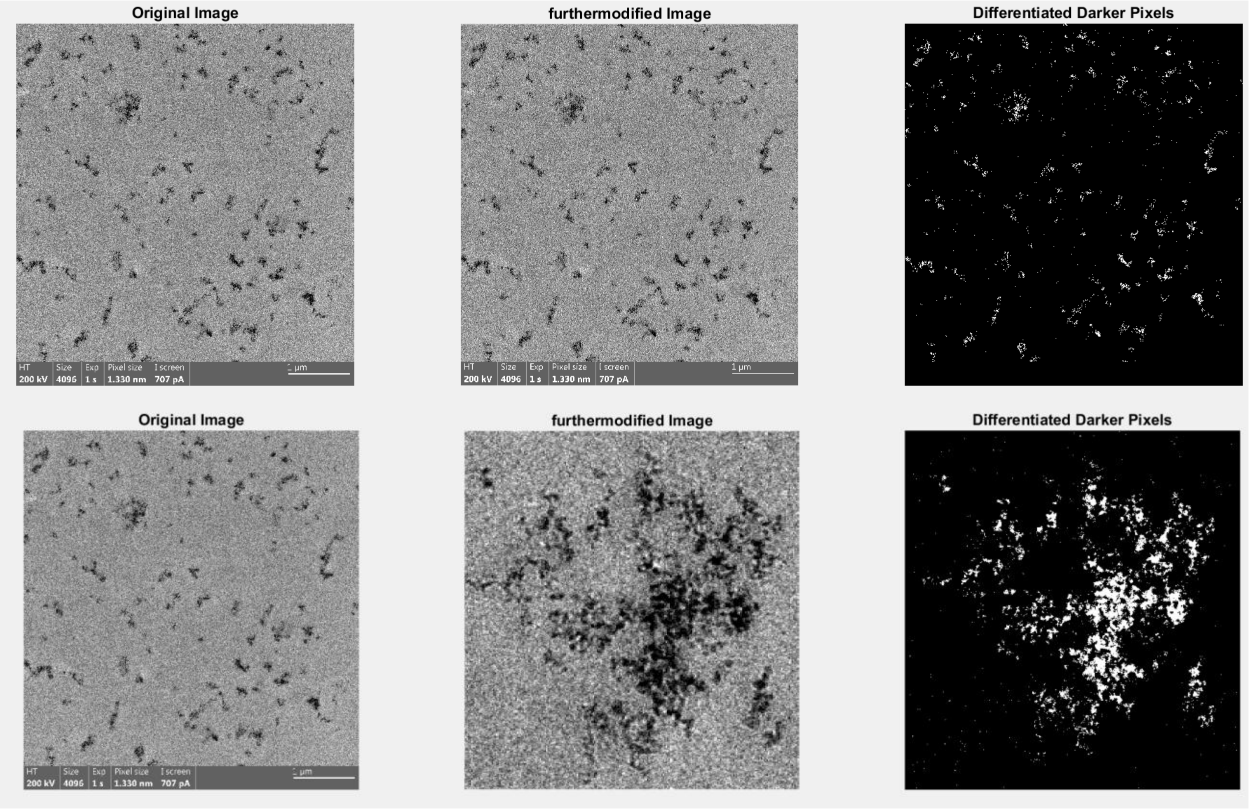

Figure S9: TEM image with threshold of 50 and no filter

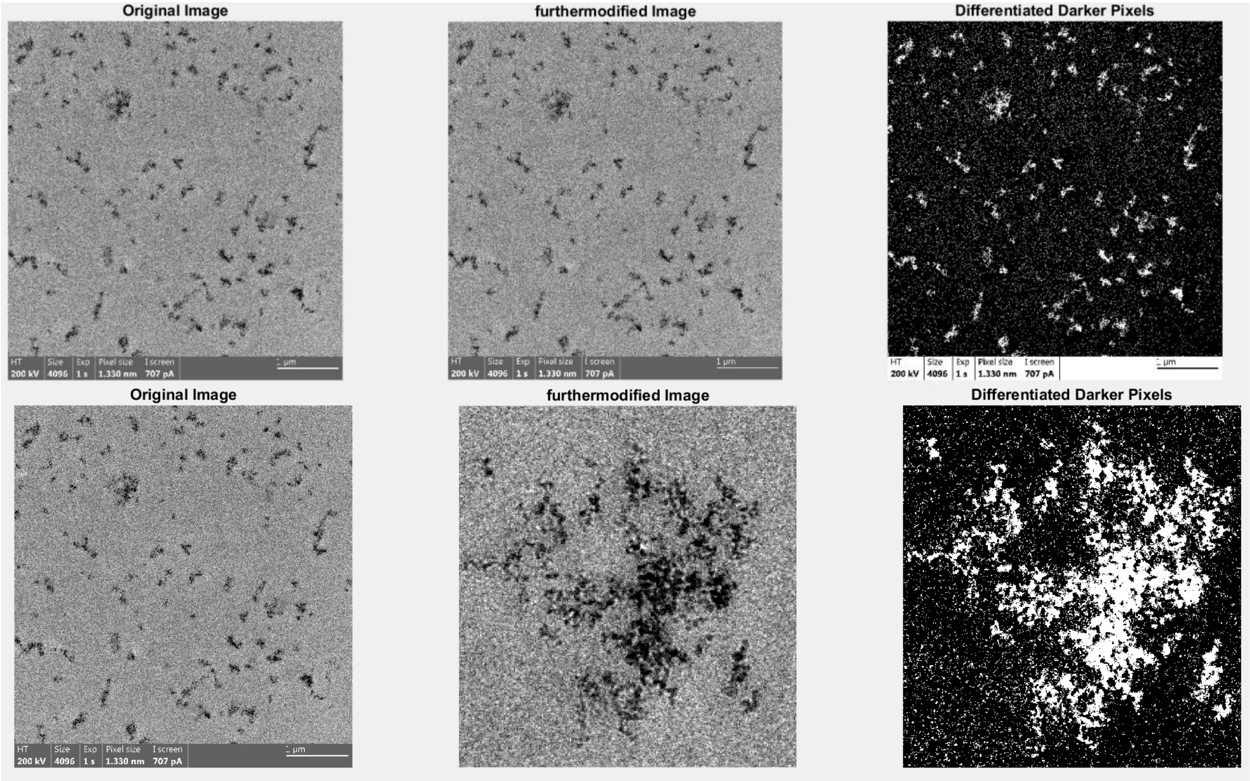

Figure S10: TEM image with threshold of 98 and no filter

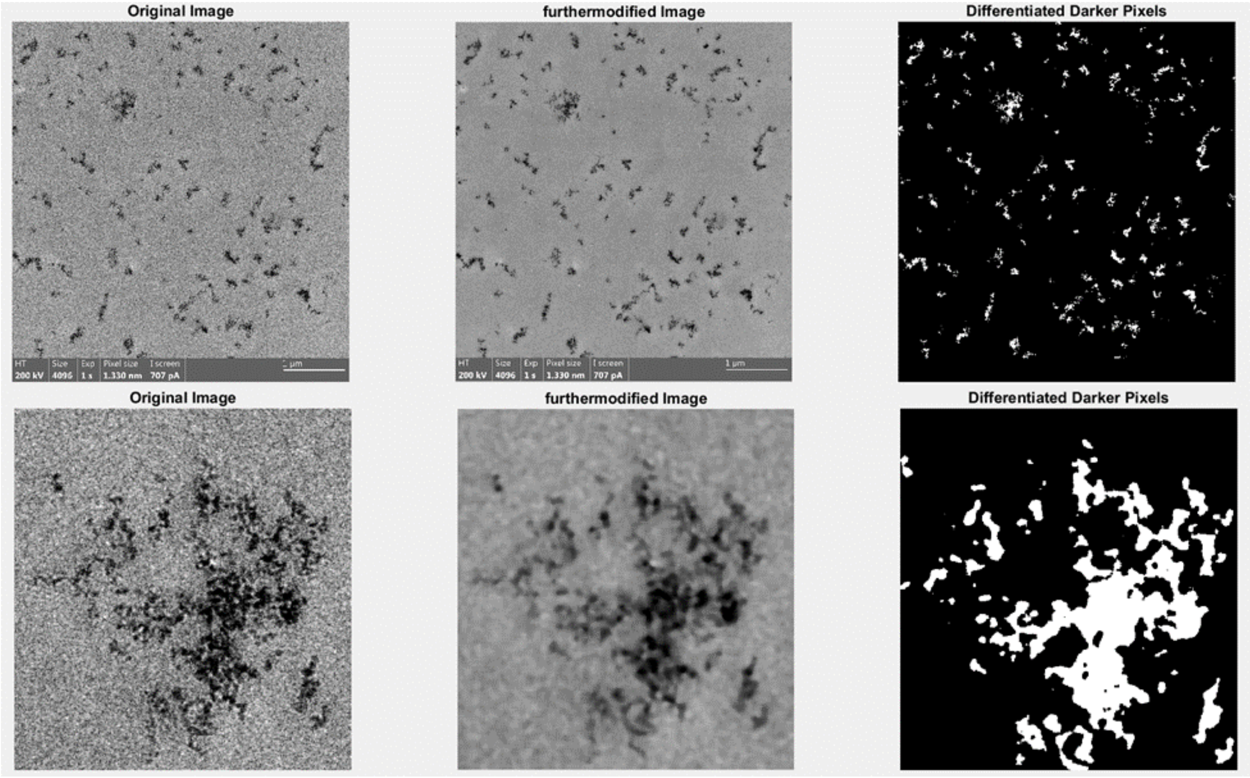

Figure S11: TEM image with threshold of 97 and filter 10

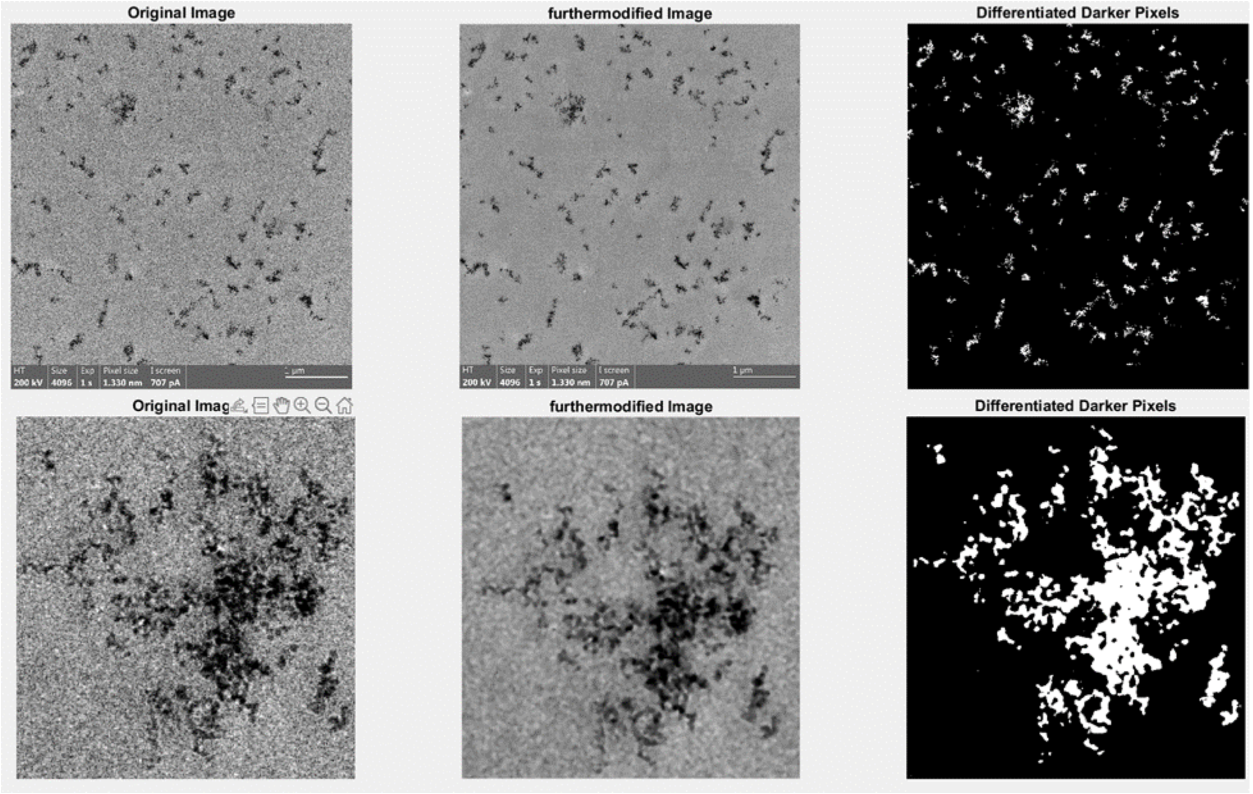

Figure S12: TEM image with threshold of 97 and filter 6

To prevent the inclusion of bottom sections during analysis and subsequent miscounting, the TEM images needed cropping. Cropping was not feasible using external software such as snipping tool due to potential resolution degradation when zoomed in. The adjusted threshold had to be tailored for each image individually due to varying background shades among the pictures. Four TEM images at 1µm were analyzed, and the average percentage of white pixels served as the "distribution percentage." Here is a picture of the MATLAB code used:

```
% Read the image
image = imread('20240226 11000 x Ceta 0002 Ceta.tif'); % Replace 'your_image.jpg' with the path to your image
% Convert the image to grayscale if it's not already in grayscale
if size(image, 3) == 3
    grayImage = rgb2gray(image);
else
    grayImage = image;
end

% Display the image
imshow(grayImage);

% Use imcrop to interactively select the region to crop
NewgrayImage = imcrop;
filtermean=9
filtered_image=medfilt2(NewgrayImage,[filtermean filtermean]);

% Define a threshold to differentiate between dark and gray pixels
threshold = 115 % You can adjust this threshold value
% Differentiate darker pixels from gray pixels
binaryImage = filtered_image < threshold;
% Display the original image and the binary image
figure;
subplot(1, 4, 1);
imshow(grayImage);
title('Original Image');
subplot(1, 4, 2);
imshow(NewgrayImage);
title('cropped Image');
subplot(1, 4, 3);
imshow(filtered_image);
title('furthermodified Image');
subplot(1, 4, 4);
imshow(binaryImage);
title('Differentiated Darker Pixels');
% Count the number of darker pixels
darkerPixels = sum(filtered_image(:) < threshold);
% Count the total number of pixels in the image
totalPixels = numel(filtered_image);
% Calculate the percentage of darker pixels
percentageDarkerPixels = (darkerPixels / totalPixels) * 100;
% Display the results
fprintf('Number of darker pixels: %d\n', darkerPixels);
fprintf('Total number of pixels: %d\n', totalPixels);
fprintf('Percentage of darker pixels: %.2f%%\n', percentageDarkerPixels);
```

Figure S13: MATLAB code for TEM image analysis

S5. Water Contact Angle Measurements

Water contact angle measurements were performed on an OCA 150 apparatus from DataPhysics Instruments GmbH. The setup was equipped with a 0.5 mL GASTIGHT #1750 syringe that deposited 5 μL droplet of Milli-Q water at a rate of 0.5 μL/s. The SCA20 software was used to determine the mean contact angle for each drop. Each sample was tested at five different locations on the film and the results were averaged to give a single mean value for the contact angle. The contact angle was captured at 5 seconds and again at 2 minutes, which was chosen as the stop time since this was the point where the evaporation of the water was visible through the camera and outpaced the flattening of the water drop. Further, this second value was measured since the

hydrophilic nature of the samples caused the water drop to flatten, which caused the angle measured to change continuously. An example of the contact angle continuously being measured is shown in Figure S14.

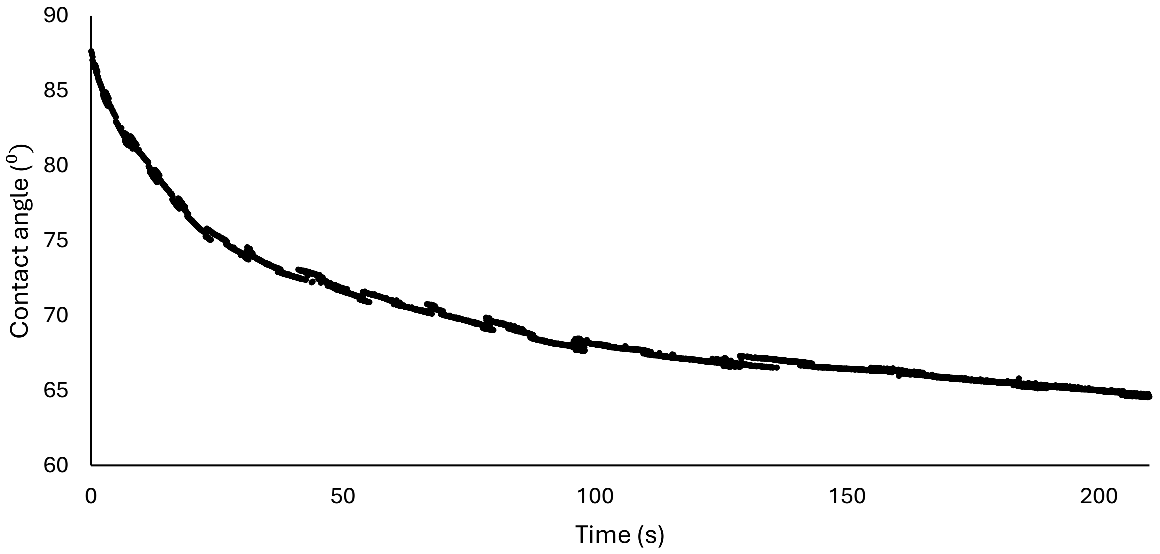

Figure S14: Contact angle vs time

The hydrophobicity of the synthesized composites was evaluated using contact angle measurements with water. After 5 seconds and 2 minutes, measurements were taken. Results in Table S1 demonstrate that the composites are hydrophilic, as their angles (76.9°-51.6°) fall below the 90° threshold. Over time, the average contact angle reduced by 14.0%, with a range of 8.6% to 27.0%. The more acute the contact angle, the greater the hydrophobicity; thus, composites above 90° are hydrophobic. Measurements could not be taken on samples containing 1wt% and 6wt% fumed silica due to their uneven surfaces impeding water drop shape maintenance. The data collected is presented below in Table S1.

Table S1: Contact angle measurements

	**Contact angle after 5s**		**Contact angle after 2min**	
	**Average**	**Stand. Dev.**	**Average**	**Stand. Dev.**
**G_0wt%**	76.90	4.20	70.27	2.81
**G_FS_3.5wt%**	51.57	2.51	37.53	2.52
**G_AS_1wt%**	72.04	3.75	62.93	1.98
**G_AS_3.5wt%**	51.63	2.65	42.22	2.60
**G_AS_6wt%**	61.24	2.96	51.92	4.00

According to Table S1, all analyzed composites are hydrophilic since their measured contact angles (<90°) fall below the 90° threshold. The addition of a filler leads to a decrease in contact angle and enhanced hydrophilicity since both fillers are hydrophilic. Notably, the 6 wt% amino silica sample may have experienced an increase in contact angle due to poorer dispersion of fillers.

S6. Viscosity Measurement of Amine terminated PHU

The APHU's viscosity was assessed over shear rates from 0.1/s to 10/s, as presented in Figure S15. Within this range, the viscosity remained relatively steady, indicating a Newtonian fluid behavior for the APHU within the examined shear rate spectrum.

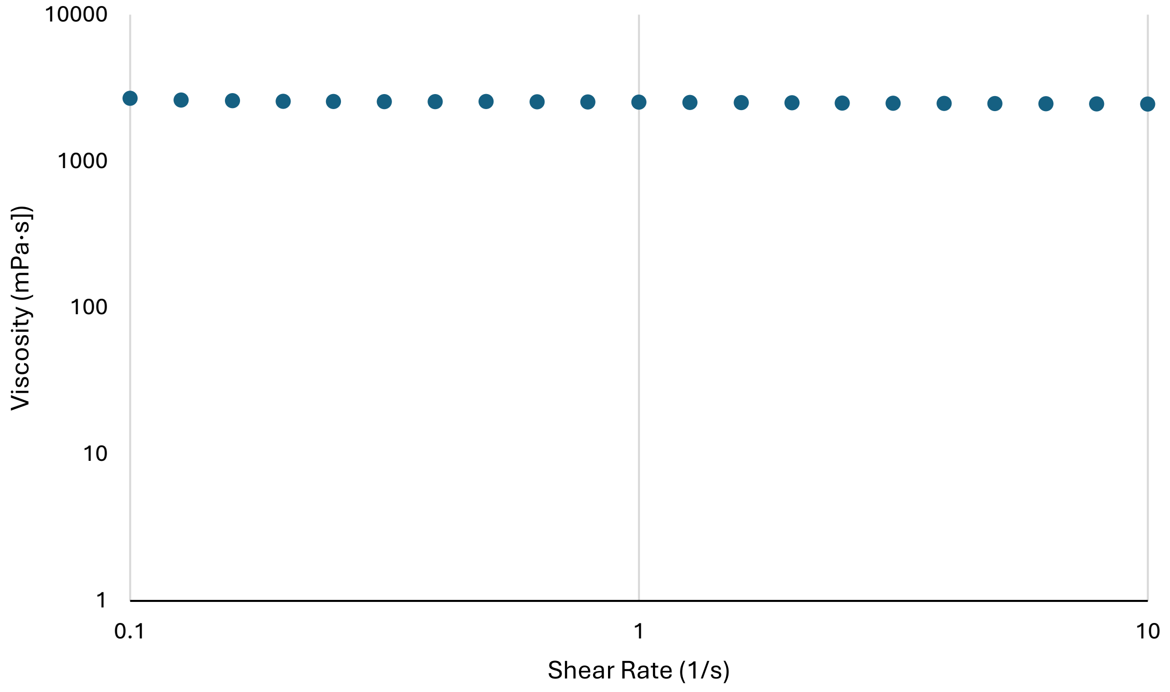

Figure S15: Viscosity of amine terminated PHU Prepolymer for different shear rates

S7. Additional Tables and Figures

Table S2: Default values for NMR test parameters

Parameter	Value (unit)
**Spectral width**	19.98.79ppm
**Selective NOE peak to irradiate**	1ppm
**NOE mixing time**	0.8s
**Centre of spectrum**	6.175ppm
**Centre of indirect dimension spectrum**	6.175ppm
**3JH coupling for HMBC**	1
**Relaxation delay**	1s
**Size of fid**	65536F1
**Number of peaks to suppress in DOUBLESOLVSUPP**	1

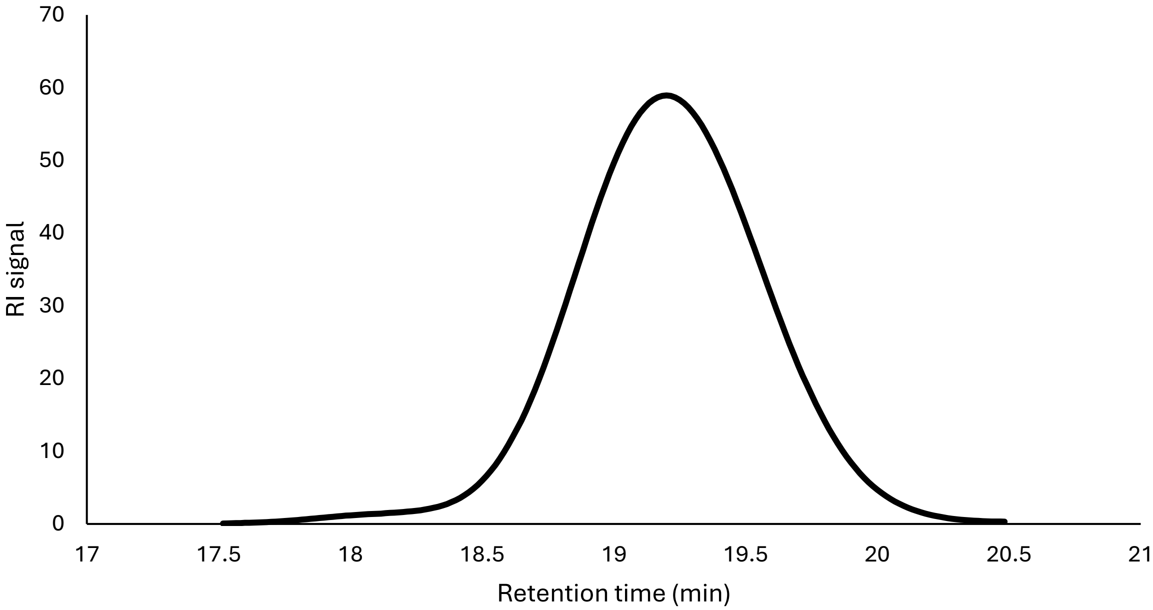

Figure S16: GPC trace of pure Jeffamine D-2000 (M_n of 3600, M_w of 3800, and Đ of 1.054)

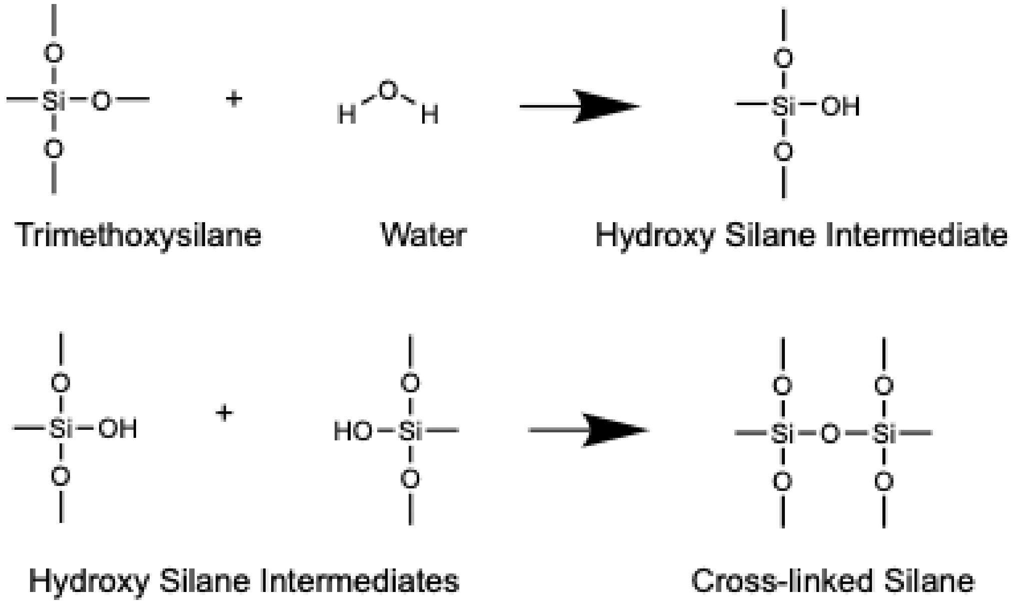

Figure S17: Silane coupling agent curing mechanism

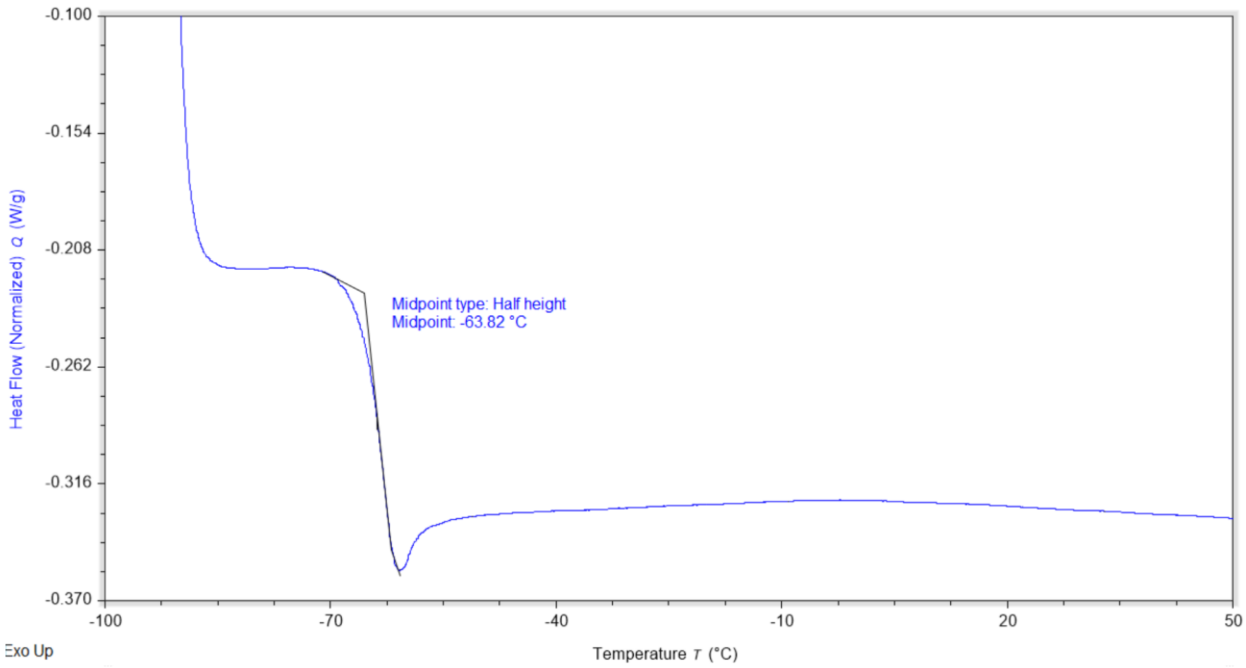

Figure S18: 2nd heating ramp of APHU for DSC analysis

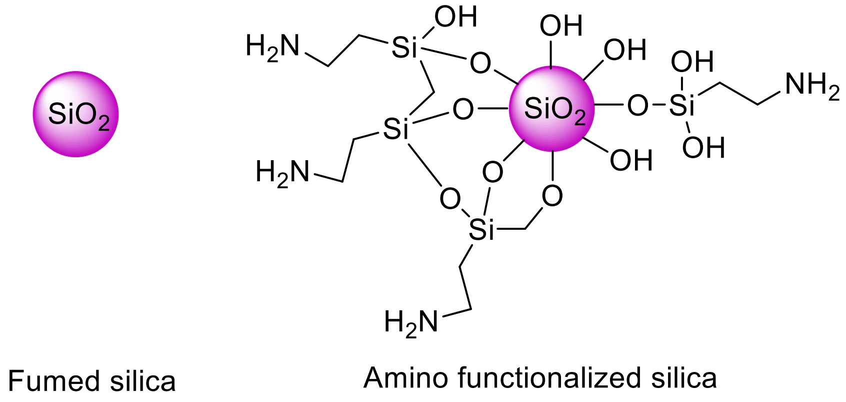

Figure S19: Visual depiction of fillers

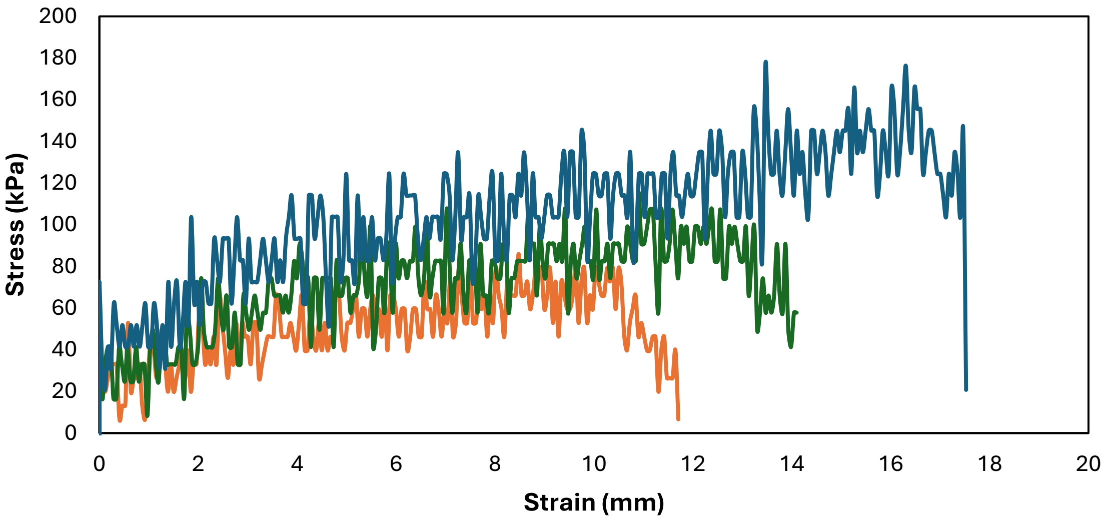

Figure S20: Graph of the 3 tensile tests
